# Role of the circRNA_34414/miR‐6960a‐5p/SIRT3 axis in postoperative delirium via CA1 Vglut1+ neurons in older mice

**DOI:** 10.1111/cns.14902

**Published:** 2024-08-13

**Authors:** Hai‐Bi Wang, Qiang Liu, Yan‐Ping Liu, Wei Dong, Jie Wan, Xin‐Hao Jiao, Yu‐Qing Wu, Tian‐Zuo Li, Hui‐Hui Miao

**Affiliations:** ^1^ Jiangsu Province Key Laboratory of Anesthesiology/NMPA Key Laboratory for Research and Evaluation of Narcotic and Psychotropic Drugs Xuzhou Medical University Xuzhou China; ^2^ Department of Anesthesiology Qidong People's Hospital/Qidong Liver Cancer Institute/Affiliated Qidong Hospital of Nantong University Nantong China; ^3^ Department of Anesthesiology, Beijing Shijitan Hospital Capital Medical University Beijing China

**Keywords:** ceRNA, circRNA, older mice, postoperative delirium, SIRT3

## Abstract

**Aims:**

Postoperative delirium (POD) is a common neurological complication in elderly patients after anesthesia/surgery. The main purpose of this study is to explore the effect of circRNA‐targeted miRNA regulating SIRT3 on mitochondrial function through ceRNA mechanism under the surgical model of tibial fracture and to further explore the potential mechanism of postoperative delirium mediated by circRNA, so as to provide new ideas for clinical diagnosis and prevention of POD.

**Methods:**

The surgical model of tibial fracture under sevoflurane anesthesia caused acute delirium‐like behavior in elderly mice. We observed that the decrease of SIRT3 and mitochondrial dysfunction was related to POD, and miRNA and circRNA (circRNA_34414) related to SIRT3 were further studied. Through luciferase and RAP, we observed that circRNA_34414, as a miRNA sponge, was involved in the regulation of SIRT3 expression.

**Results:**

Postoperative delirium in elderly mice showed decreased expression of hippocampal circRNA_34414, increased expression of miR‐6960‐5p, decreased expression of SIRT3, and impaired mitochondrial membrane potential. Overexpression of circRNA_34414, or knockdown of miR‐6960‐5p, or overexpression of SIRT3 in hippocampal CA1 glutamatergic neurons significantly upregulated hippocampal SIRT3 expression, increased mitochondrial membrane potential levels, and significantly ameliorated postoperative delirium in aged mice; CircRNA_34414 ameliorates postoperative delirium in mice, possibly by targeting miR‐6960‐5p to upregulate SIRT3.

**Conclusions:**

CircRNA_34414 is involved in the improvement of postoperative delirium induced by anesthesia/surgery by upregulating SIRT3 via sponging miR‐6960‐5p.

## Graphical abstract

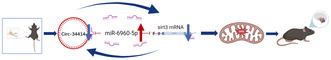

## 1 INTRODUCTION

Postoperative delirium (POD) is a mental disorder characterized by rapid changes and fluctuations in attention, awareness, and cognition after anesthesia and surgery.^1^, ^2^ POD often occurs in seniors, with its occurrence ranging from 8% to 70% depending on the factors such as the specific surgical procedure. The occurrence of POD prolonged the hospital stay. It was reported that the 30‐day mortality rate of POD was 7%–10%, whereas that without delirium was 1%.^3^, ^4^ There was a strong correlation between the presence of POD and a notable increase in medical expenses.^5^ Therefore, comprehensive study of the mechanism of POD, looking for specific biomarkers and the development of targeted therapeutic drugs has important clinical and social significance.

Current research suggests that the pathological and physiological mechanisms of POD are not fully understood and are mainly believed to be related to oxidative stress, neurotransmitters and neuroinflammation.^6^, ^7^ Mitochondria are known to have a significant impact on neurodegenerative diseases associated with the aging process.^8^ SIRT3 is a nicotinamide adenine dinucleotide (NAD) + −dependent III histone deacetylase.^9^, ^10^ SIRT3 deacetylation regulates mitochondrial function and is associated with numerous age‐related diseases, such as cardiovascular disease and neurodegenerative diseases.^8^ SIRT3 regulates mitochondrial function by modulating energy metabolism, ROS production and clearance, the electron transport chain, the mitochondrial membrane permeability transport channels, mitochondrial dynamics, etc.^11^, ^12^, ^13^, ^14^

Circular RNAs (circRNAs) represent a recently identified category of non‐coding RNAs (ncRNAs). Unlike other linear RNAs, circRNA is a covalent closed‐loop structure without the traditional 5′ and 3′ ends, which is conducive to improving its stability.^15^ Recent research indicates that circRNA may be involved in several physiological or pathological processes and hold an important role in these processes.^16^, ^17^, ^18^ Furthermore, there is evidence linking circRNA to neurological disorders such as Alzheimer's disease (AD).^19^, ^20^, ^21^

MicroRNA (miRNA) is about 22 nucleotides long and regulates gene expression at the post‐transcriptional level in a sequence‐specific manner.^22^ It has been demonstrated that the inhibition of miRNAs is cross‐regulated by competitive endogenous RNAs (ceRNAs) that carry the same miRNA response elements (MRE) that isolate miRNAs.^23^ This concept led to the development of complex ceRNA networks (ceRNETs), highlighting the complex role of miRNAs in regulating a wide range of physiological and pathological processes.

In summary, this project intends to demonstrate whether circRNA‐targeted miRNA regulation of SIRT3 can alleviate the occurrence and development of POD caused by anesthesia/surgery, to provide a theoretical basis for elucidating the pathogenesis of POD and developing new prevention and treatment drugs.

## 2 MATERIALS AND METHODS

### 2.1 Animals and materials

In this experiment, 18‐month‐old male C57 mice and Vglut‐1 mice were used with a weight of 22–28 g. All animals were kept in an animal house with a constant temperature of 24 ± 2°C and a constant humidity of 40%–60%, and the circadian rhythm of 12 h/12 h. All animal experiments were approved by the Laboratory Animal Ethics Committee of Xuzhou Medical University, with ethical approval number (IACUC: 202204A190). All animal experiments were complied with the ARRIVE guidelines and carried out in accordance with the National Research Council's Guide for the Care and Use of Laboratory Animals. The detailed materials and methods of this study are found in Supporting Information.

### 2.2 Statistical analysis

The data were analyzed using GraphPad Prism 8.0 (GraphPad Software, Inc.). Shapiro–Wilk test was used to check whether the data were normally distributed and all the data followed a normal variable distribution. The results are shown as the mean ± SEM if they were normally distributed, or as median (interquartile range) for categorical variables. The unpaired, 2‐tailed *t*‐test or Mann–Whitney *U* test was used to compare the differences between the two groups. To calculate the differences among the four groups, one‐way ANOVA was performed. Two‐way ANOVA followed by Tukey's multiple‐comparison test was used to compare how two factors affected a numeric result. The threshold for significance was set at *p* < 0.05.

## 3 RESULTS

### 3.1 Anesthesia and surgery induced delirium‐like behaviors in older mice

**FIGURE 1:**
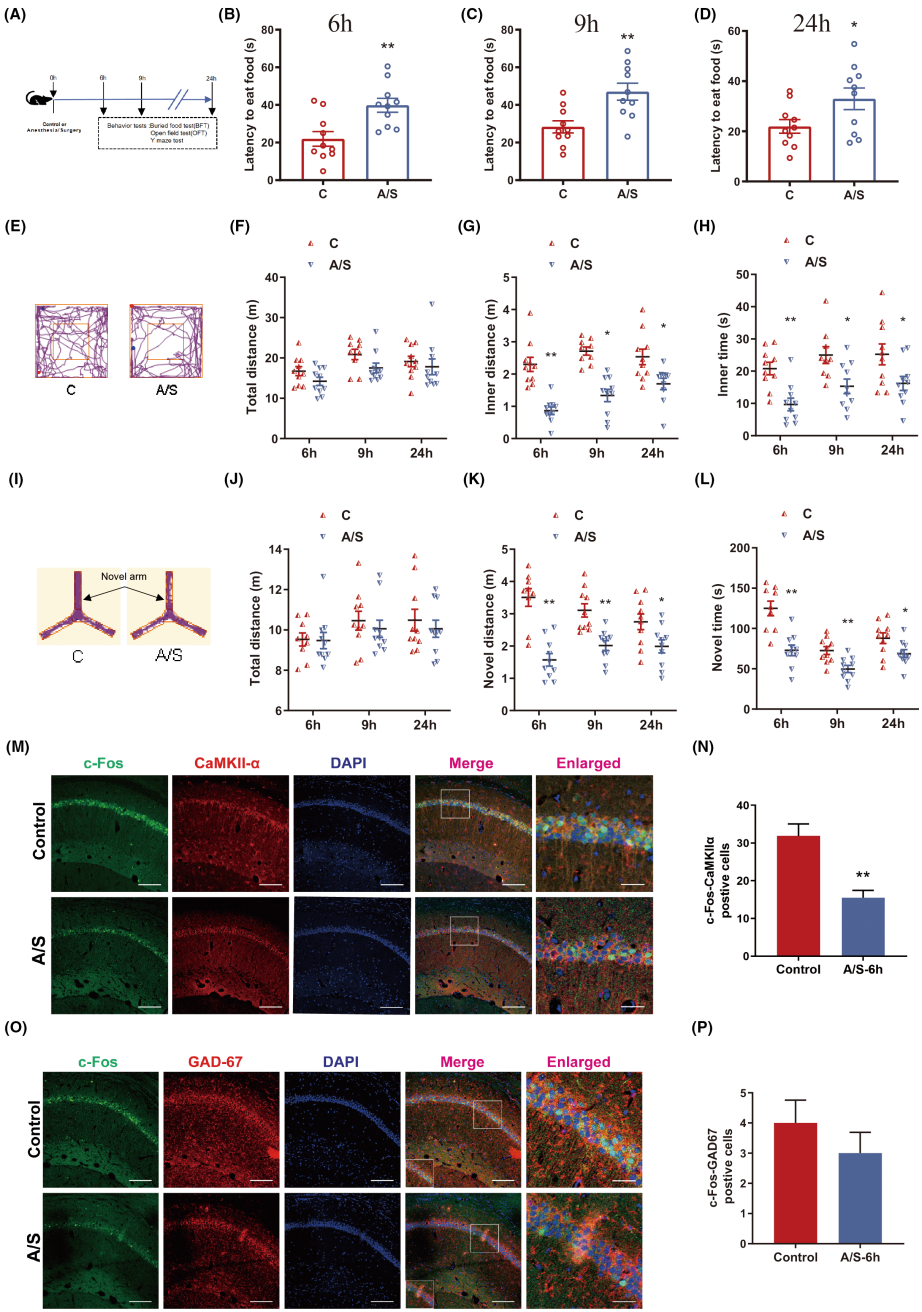
Anesthesia/surgery induced delirium‐like behaviors and decreased the excitability of glutamatergic neurons. (A) Schematic diagram of behavioral tests. (B,D) The latency to eat food in bury food test at 6, 9, and 24 h postoperatively. (B, 6 h: *N* = 10, *p* < 0.01, C vs. A/S, *t*‐test; C, 9 h: *N* = 10, *p* < 0.01, C vs. A/S, *t*‐test; D, 24 h: *N* = 10, *p* = 0.04, C vs. A/S, *t*‐test.) (E) Representative tracking of exploration paths in open field test. (F) The total distance in the open field test at 6, 9, and 24 h postoperatively (6 h: *N* = 10, *p* = 0.11, C vs. A/S, *t*‐test; 9 h: *N* = 10, *p* = 0.17, C vs. A/S, *t*‐test; 24 h: *N* = 10, *p* = 0.59, C vs. A/S, *t*‐test). (G) The distance traveled in the inner zone of the open field test (6 h: *N* = 10, *p* < 0.01, C vs. A/S, *t*‐test; 9 h: *N* = 10, *p* = 0.02, C vs. A/S, *t*‐test; 24 h: *N* = 10, *p* = 0.012, C vs. A/S, *t*‐test). (H) The time spent in the inner zone of the open field test (6 h: *N* = 10, *p* < 0.01, C vs. A/S, *t*‐test; 9 h: *N* = 10, *p* = 0.014, C vs. A/S, *t*‐test; 24 h: *N* = 10, *p* = 0.03, C vs. A/S, *t*‐test). (I) Representative tracking of exploration paths in the Y maze test. (J) The total distance traveled in the Y maze (6 h: *N* = 10, *p* = 0.96, C vs. A/S, *t*‐test; 9 h: *N* = 10, *p* = 0.53, C vs. A/S, *t*‐test; 24 h: *N* = 10, *p* = 0.54, C vs. A/S, *t*‐test). (K) The distance traveled in the novel arms of the Y maze (6 h: *N* = 10, *p* < 0.01, C vs. A/S, *t*‐test; 9 h: *N* = 10, *p* < 0.01, C vs. A/S, *t*‐test; 24 h: *N* = 10, *p* = 0.03, C vs. A/S, *t*‐test). (L) The time spent in the novel arms of the Y maze (6 h: *N* = 10, *p* < 0.01, C vs. A/S, *t*‐test; 9 h: *N* = 10, *p* < 0.01, C vs. A/S, *t*‐test; 24 h: *N* = 10, *p* = 0.03, C vs. A/S, *t*‐test). (M) Representative images of c‐Fos/CaMKIIα/DAPI immunofluorescence in CA1 region at 6 h postoperatively (scale bar = 100 μm, left; scale bar = 50 μm, right). (N) The number of c‐Fos^+^/CaMKIIα^+^ cells (*n* = 6, *p* < 0.01, Control vs. A/S‐6 h, *t*‐test). (O) Representative images of c‐Fos/GAD67/DAPI immunofluorescence in CA1 region at 6 h postoperatively (scale bar = 100 μm, left; scale bar = 50 μm, right). (P) The number of c‐Fos^+^/GAD67^+^ cells (*n* = 6, *p* = 0.35, Control vs. A/S‐6 h, *t*‐test). The data are plotted as Mean ± SEM. *p < 0.05, **p < 0.01 compared with the control group.

Buried food, open field and Y maze test were used to evaluate postoperative delirium at 6, 9 and 24 h after anesthesia/surgery (Figure 1A). First, the latency to eat food were significantly increased in A/S group at 6, 9 and 24 h postoperatively (Figure 1B–D). Second, in the open field test, the distance (Figure 1G) and the time (Figure 1H) in the inner zone were significantly decreased. Third, in the Y‐maze test, the distance (Figure 1K) and the time (Figure 1L) spent in the novel arm was lower. However, there was no significant difference in the total distance in OFT (Figure 1E,F) and Y maze test (Figure 1I,J). Therefore, the behaviors tests indicated that anesthesia and surgery caused postoperative delirium in older mice.

### 3.2 Vglut1+ neurons participate in postoperative delirium of older mice

To further investigate whether the neuron type specific is involved in POD, we detected the co‐expression of c‐Fos with CaMKIIα or GAD67 in the CA1 region by immunofluorescence in older mice. The number of c‐Fos/CaMKIIα positive cells but not c‐Fos/GAD67 positive cells was significantly reduced in the A/S group (Figure 1M–P).

Vglut1‐cre mice was used and the electrophysiological recordings were made in the CA1 region (Figure S1A). The whole‐cell patch‐clamp recording results showed that the threshold current of induced action potential increased significantly in the A/S group of Vglut1 mice (Figure S1B), firing rate of action potentials was significantly decreased (Figure S1C,D), and the resting membrane potential did not change significantly (Figure S1E). These results suggested that anesthesia/surgery reduced the Vglut1 neuronal activity.

For optogenetics, we used AAV‐DIO‐ChR2‐eGFP or a control (green fluorescent protein [GFP]) vector in Vglut1+ neurons (Figure S1H,P), the results showed that blue light (473 nm) activated robust firing in the A/S group mice (Figure S1F). In addition, photoactivation significantly shortened the latency to eat food in the buried food test in the A/S group of Vglut1 mice (Figure S1I). In the open field test, the distance and time of mice spent in the inner zone were rescued (Figure S1K,L). In the Y‐maze test, the distance and time in the novel arm were significantly longer (Figure S1N,O). In addition, there was no significant difference in the total distance in the open field and Y maze test (Figure S1J,M). These results showed that Vglut1 neurons activation in the CA1 region could protect the postoperative delirium behavior of older mice.

Correspondingly, yellow light (589 nm) was used to inhibit Vglut1+ neurons in the CA1 region in the control group mice, the action potential was effectively suppressed (Figure S1G). In the meanwhile, the latency to eat food was significantly prolonged in the control group of Vglut1 mice (Figure S1Q). In the open field test, the distance and time in the inner zone were significantly decreased (Figure S1S,T); Similarly, in the Y‐maze test, the distance and time in the novel arm was significantly shortened (Figure S1V,W). In addition, there was no significant difference in the total distance in the open field and Y maze test (Figure S1R,U). These results showed that optogenetic inhibition of Vglut1 neurons in the CA1 region could lead to delirium‐like behaviors in naïve older mice.

Then, we used chemogenetics to activate Vglut1 neurons with AAV‐hM3Dq virus injection. Immunofluorescence results showed that the intraperitoneal injection of CNO could significantly increase c‐Fos expression in the A/S group mice (Figure S2A). CNO injection also increased the frequency of action potentials in the current clamp mode of CA1 slice (Figure S2B). The behavioral results showed that CNO injection could significantly shorten the latency to eat food in the buried food test in the A/S group Vglut1 mice (Figure S2C). The distance and time to enter the inner zone of the open field test was rescued (Figure S2E,F) and the distance and time spent in the novel arm of the Y maze test was prolonged (Figure S2H,I). In addition, there was no obvious difference in the total distance in the open field and Y maze test (Figure S2D,G). Next, chemogenetics was used to inhibit Vglut1 neurons with AAV‐hM4Di virus injection in the control group mice. Immunofluorescence results showed that the injection of CNO could significantly reduce the c‐Fos expression (Figure S2J). CNO injection reduced the frequency of action potential (Figure S2K). The behavioral results showed that CNO injection significantly prolonged the latency to eat food in the buried food test (Figure S2L). The distance and time to enter the inner zone of the open field test was reduced (Figure S2N,O) and the distance and time spent in the novel arm was shortened (Figure S2Q,R). There was no obvious difference in the total distance in the open field and Y maze test (Figure S2M,P).

### 3.3 Anesthesia/surgery induced SIRT3 expression decreasing and mitochondrial dysfunction

The SIRT3 protein expression was significantly reduced at 6, 9 and 24 h after anesthesia and surgery (Figure S3A–C), and the SIRT3 fluorescence expression in the CA1 area was also reduced specific in the CaMKIIα+ but not GAD67^+^ neurons at 6 h after anesthesia and surgery (Figure S4A–D). There was no significant difference in SOD2 protein level between groups (Figure S3D–F), whereas the AC‐SOD2 expression was increased in A/S group (Figure S3G–I). The MMP level was reduced significantly (Figure S3J–L).

### 3.4 Specific overexpress SIRT3 in Vglut1 neuron improved postoperative delirium in older mice

**FIGURE 2:**
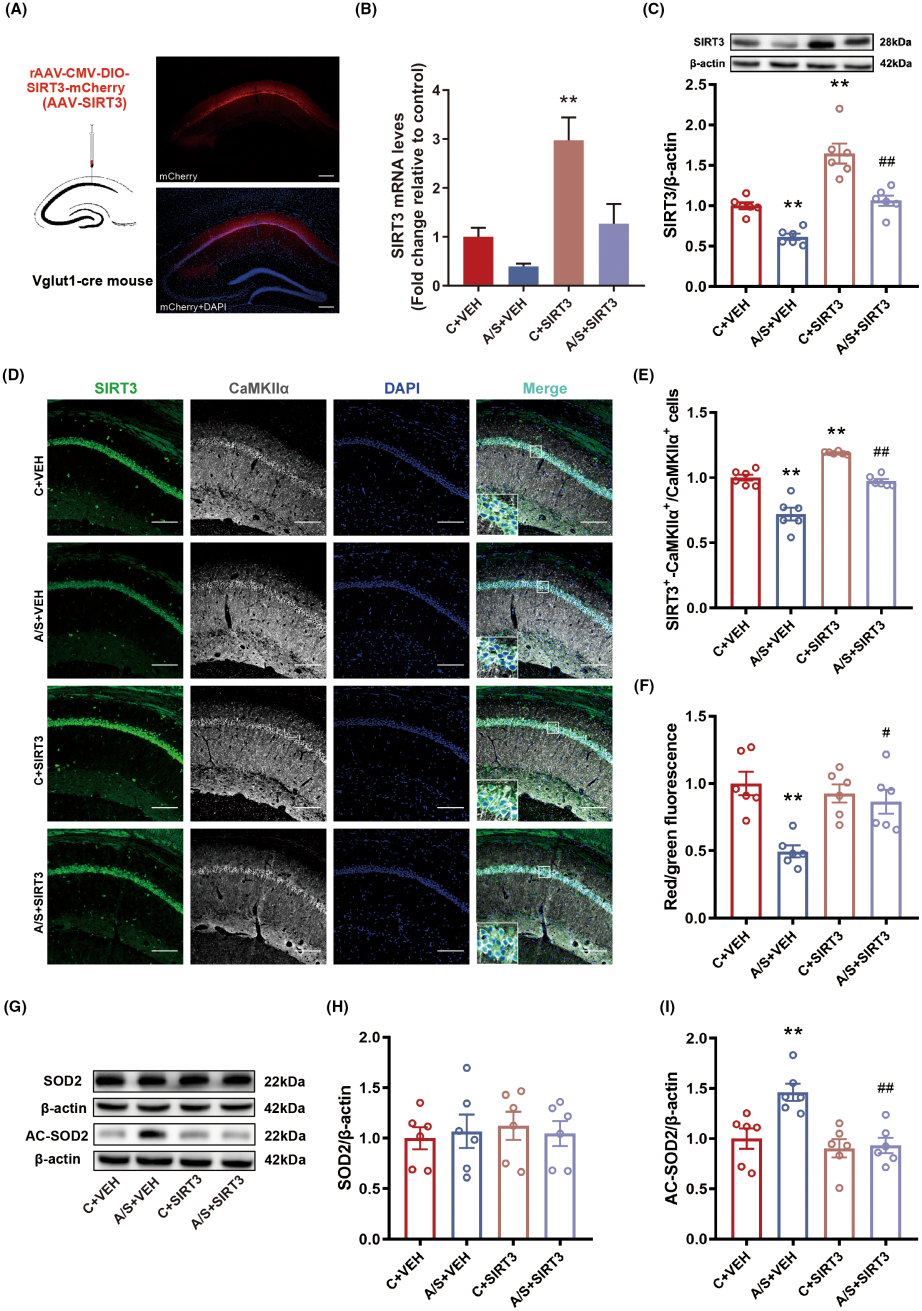
Vglut1‐specific overexpression of SIRT3 improved the postoperative mitochondrial dysfunction. (A) The location and schematic for viral microinjection with a rAAV‐CMV‐DIO‐SIRT3‐mCherry (AAV‐SIRT3) or a control rAAV‐CMV‐DIO‐ mCherry vehicle (AAV‐VEH). (B) The expressions of SIRT3 mRNA in hippocampal CA1 region among the four groups (*n* = 6, *F*(3,20) = 11.73, *p* = 0.0014, One‐way ANOVA). (C) Protein levels of SIRT3 were estimated by Western Blot (*n* = 6, *F*(3,20) = 31.93, *p* < 0.01, One‐way ANOVA). (D) Representative images of CaMKIIα+ and SIRT3 staining (Scale bar: 100 μm) in hippocampal CA1 region of left. (E) The percentage of SIRT3 and CaMKIIα+ co‐labeling cells in hippocampal CA1 region (*n* = 6, *F*(3,20) = 45.08, *p* < 0.01, One‐way ANOVA). (F) The levels of MMP in the hippocampal mitochondria (*n* = 6, *F*(3,20) = 9.04, *p* < 0.01, One‐way ANOVA). (G–I) Protein levels of SOD2 and AC‐SOD2 were estimated by Western Blot (H: *N* = 6, *F*(3,20) = 0.14, *p* = 0.94, One‐way ANOVA; I: *N* = 6, *F*(3,20) = 8.83, *p* < 0.01, One‐way ANOVA). The data are plotted as Mean ± SEM. *p < 0.05, **p < 0.01 compared with the C + VEH group. ^#^ *p* < 0.05, ^##^ *p* < 0.01 compared with the A/S + VEH group.

To verify the role of SIRT3 in POD, we injected rAAV‐SIRT3 virus in the CA1 region of Vglut1‐cre mice (Figure 2A), which significantly reversed the reduced SIRT3 mRNA (Figure 2B) and protein levels (Figure 2C), and significantly increased the expression of SIRT3 in CaMKII⍺^+^ neurons (Figure 2D–E). Overexpression of SIRT3 reversed MMP level (Figure 2F) and AC‐SOD2 expression (Figure 2G–I). SIRT3 overexpression could also reverse the activity of Vglut1+ neuron (Figure S4E), which was manifested by a significant decrease in the threshold current (Figure S4F) and a significant increase in the frequency of action potential release (Figure S4G). There was no significant change in resting membrane potential (Figure S4H). At the same time, SIRT3 overexpression rescued the latency to eat food (Figure S4I). In the open field test, the distance and time in the inner zone was significantly increased (Figure S4L–M); the distance and time spent in the novel arm was significantly increased in the Y maze test (Figure S4P–Q). In addition, there was no significant difference in the total distance in the open field and the Y maze test (Figure S4J–K,N–O).

### 3.5 CircRNA_34414 targets miR‐6960‐5p to regulate SIRT3 expression

Our previous circRNA microarray study revealed 36 of 124 circRNAs significantly decreased (>1.5‐fold) (GEO accession number GSE165798; https://www.ncbi.nlm.nih.gov/geo/query/acc.cgi?acc=GSE165798). CircRNA _34414 was the only possible circRNA that might regulate SIRT3 expression by bioinformatics analysis. CircRNA‐microRNA interactions were predicted using microRNA predicted software based on TargetScan and miRanda (Arraystar Corporation), only the miRNA response element (MRE) miR‐6960‐5p of circRNA_34414 could bind to the 3′‐UTR of SIRT3 mRNA (Figure S5A,B).

Consistent with the microarray data, the expression of circRNA_34414 and SIRT3 mRNA in the A/S group was significantly reduced (Figure S5C,E), and the expression of miR‐6960‐5p was significantly increased (Figure S5D). To verify that miR‐6960‐5p can bind directly to circRNA_34414 and SIRT3, we performed a Dual‐Luciferase Reporter Assay. MiR‐6960‐5p significantly reduced the luciferase activity of circRNA_34414‐wt and SIRT3‐wt reporters, but did not reduce the luciferase activity of circRNA_34414‐mut and SIRT3‐mut reporters (Figure S5F–I). Subcellular localization of circRNA_34414 was detected by FISH and nuclear plasma isolation experiments. CircRNA_34414 and miR‐6960‐5p were mostly located in the cytoplasm (Figure S5J,K). CircRNA_34414 was enriched and the miRNA‐6960‐5p was pulled down by circRNA_34414 probe (Figure S5L,M). Consistent with this finding, miR‐6960‐5p levels were not changed with circRNA_34414 overexpression (Figure S5N); circRNA_34414 levels were not significantly altered after the overexpression or inhibition of miR‐6960‐5p (Figure S5O,P). Collectively, these results demonstrate that circRNA_34414 functions as a sponge for miR‐6960‐5p.

### 3.6 Overexpression cicrRNA_34414 improved postoperative delirium in older mice

**FIGURE 3:**
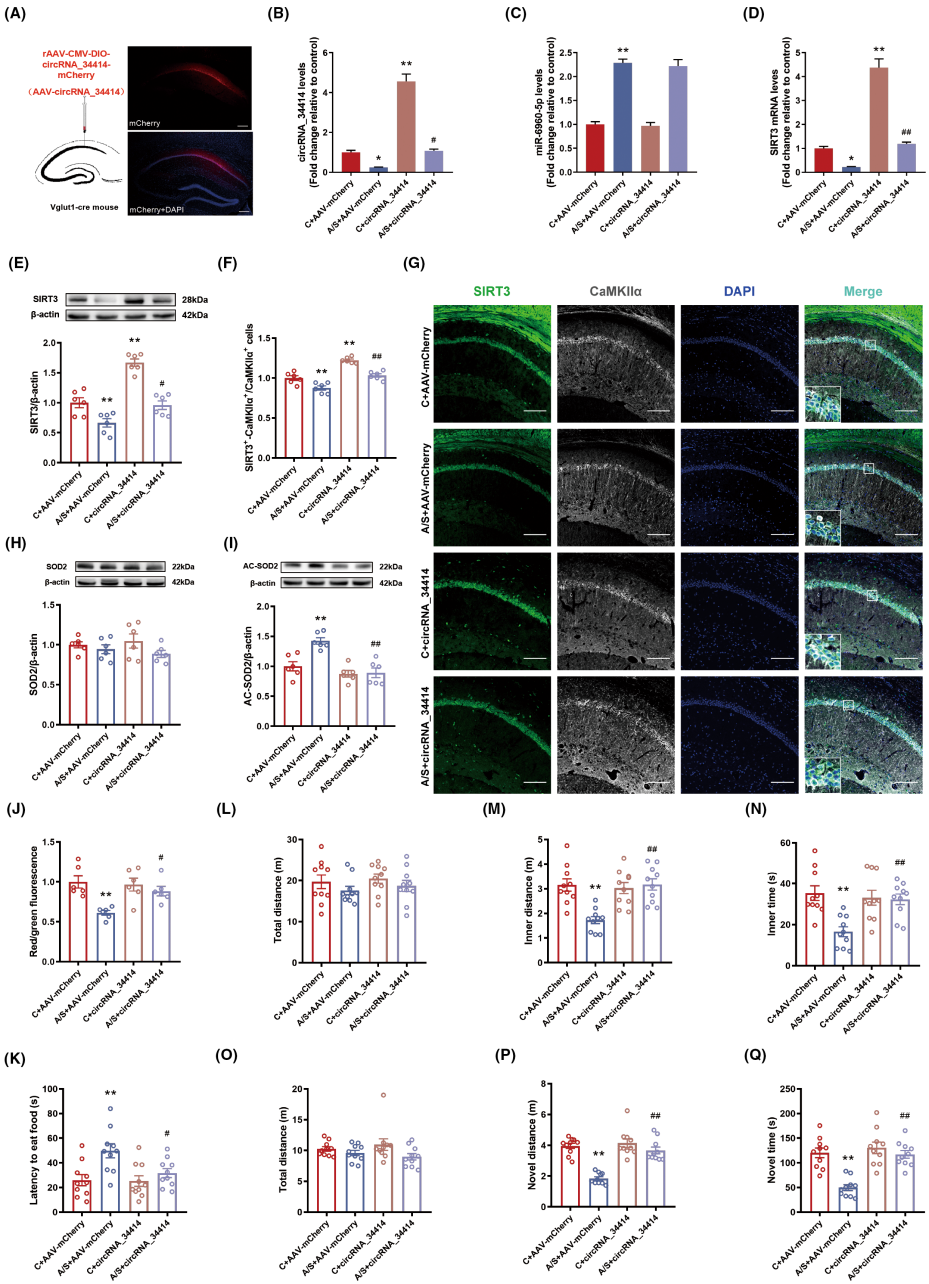
Effect of overexpression of circRNA_34414 on postoperative delirium in Vglut1 mice. (A) The location and schematic for viral microinjection with a rAAV‐CMV‐DIO‐circRNA_34414‐P2A‐mCherry (AAV‐circRNA_34414) or a control rAAV‐CMV‐DIO‐P2A‐mCherry vehicle (AAV‐mCherry). (B–D) The expressions of circRAN_34414, miR‐6960‐5p and SIRT3 mRNA in hippocampal CA1 region among the four groups (B: *N* = 6, *F*(3,20) = 103.3, *p* < 0.01, One‐way ANOVA; C: *N* = 6, *F*(3,20) = 65.13, *p* < 0.01, One‐way ANOVA; D: *N* = 6, *F*(3,20) = 92.15, *p* < 0.01, One‐way ANOVA). (E) Protein levels of SIRT3 were estimated by western blot among the four groups (*n* = 6, *F*(3,20) = 35.00, *p* < 0.01, One‐way ANOVA). (F) The percentage of co‐labeling number of SIRT3 and CaMKIIα+ in CA1 region among four groups (*n* = 6, *F*(3,20) = 37.18, *p* < 0.01, One‐way ANOVA). (G) Representative images of CaMKIIα+ stained with SIRT3 (Scale bar: 100 μm) in hippocampal CA1 region of left. (H,I) Protein levels of SOD2 and AC‐SOD2 were estimated by western blot among the four groups (H: *N* = 6, *F*(3,20) = 1.35, *p* = 0.29, One‐way ANOVA; I: *N* = 6, *F*(3,20) = 14.4, *p* < 0.01, One‐way ANOVA). (J) The levels of MMP in mitochondria among the four groups (*n* = 6, *F*(3,20) = 7.38, *p* < 0.01, One‐way ANOVA). (K) The latency to eat the food among the four groups (*n* = 10, *F*(3,36) = 6.192, *p* < 0.01, One‐way ANOVA). (L–N) The total distance, inner distance and inner time of OFT among the four groups. (L: *N* = 10, *F*(3,36) = 0.91, *p* = 0.45, One‐way ANOVA; M: *N* = 10, *F*(3,36) = 10.37, *p* < 0.01, One‐way ANOVA; N: *N* = 10, *F*(3,36) = 7.83, *p* < 0.01, One‐way ANOVA). (O–Q) The total distance, novel distance and novel time in Y maze among the four groups (O: *N* = 10, *F*(3,36) = 1.95, *p* = 0.14, One‐way ANOVA; P: *N* = 10, *F*(3,36) = 26.73, *p* < 0.01, One‐way ANOVA; Q: *N* = 10, *F*(3,36) = 16.37, *p* < 0.01, One‐way ANOVA). The data are plotted as Mean ± SEM. *p < 0.05, **p < 0.01 compared with the C + AAV‐mCherry group. ^#^ *p* < 0.05, ^##^ *p* < 0.01 compared with the A/S + AAV‐mCherry group.

We overexpressed circRNA_34414 in the CA1 region of Vglut1‐cre mice (Figure 3A), which significantly upregulated circRNA_34414 expression in A/S + circRNA_34414 group compared with A/S + AAV‐mCherry group (Figure 3B), resulting in not significant change of miR‐6960‐5p level in A/S + circRNA_34414 group compared with A/S + AAV‐mCherry group (Figure 3C) and reversed the decrease in SIRT3 mRNA (Figure 3D) and protein level caused by anesthesia/surgery (Figure 3E). Immunofluorescence results showed a significant increase of SIRT3 expression in CaMKII⍺ + neurons (Figure 3F,G). CircRNA_34414 overexpression also reversed the increase of AC‐SOD2 (Figure 3H–I) and the decrease in MMP (Figure 3J). At the same time, circRNA_34414 overexpression protected delirium‐like behavior. The latency to eat food in the buried food test (Figure 3K) was improved. The distance and time in the inner zone were significantly increased (Figure 3M,N). The distance and time spent in the novel arm were significantly increased in the Y maze (Figure 3P,Q). In addition, there was no significant difference in the total distance in the open field and Y maze test (Figure 3L,O).

### 3.7 Knockdown miR‐6960‐5p ameliorated postoperative delirium in older mice

**FIGURE 4:**
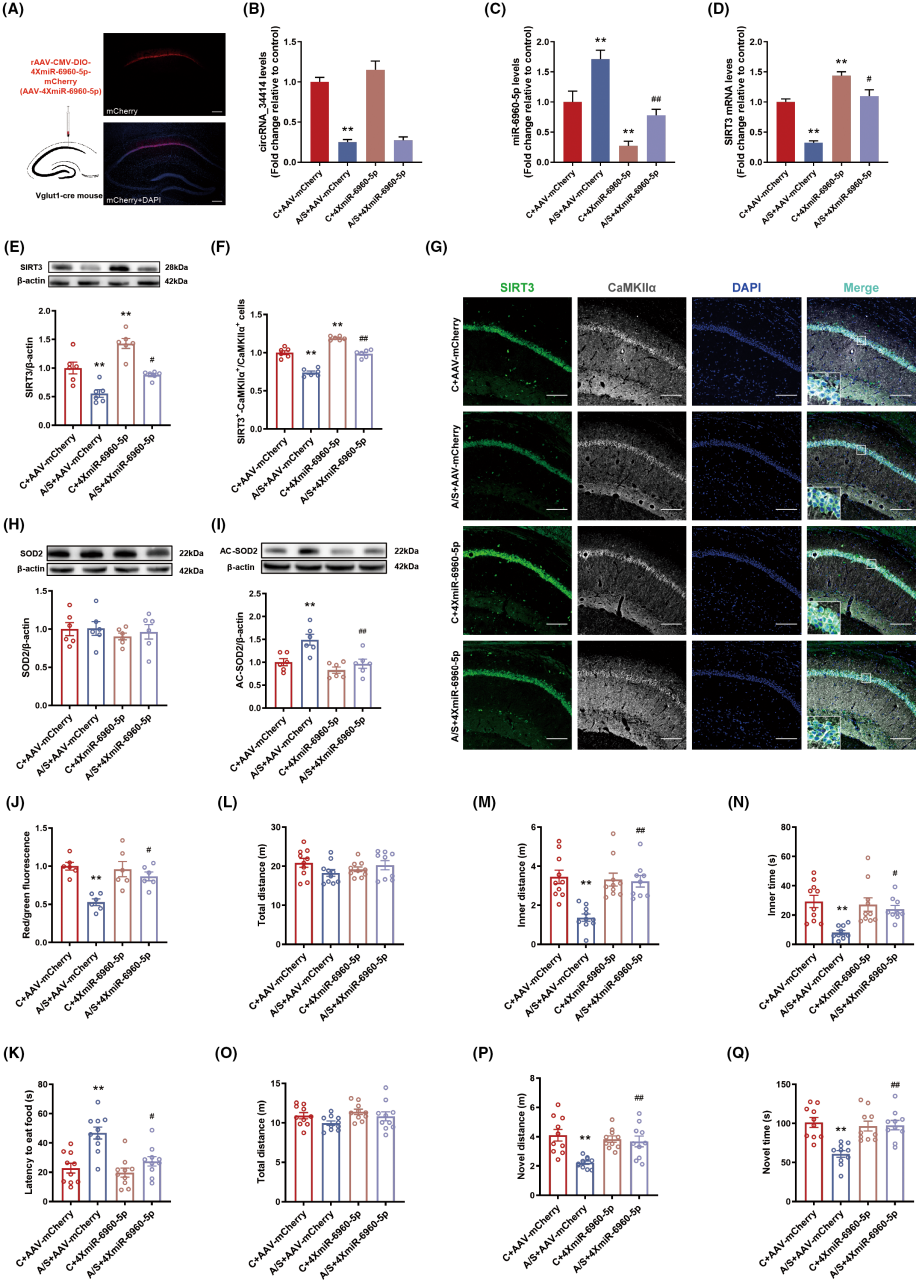
Effect of knockdown of miR‐6960‐5p on postoperative delirium in mice. (A) The location and schematic for viral microinjection with a rAAV‐CMV‐DIO‐4XmiR‐6960‐5p‐mCherry (AAV‐4XmiR‐6960‐5p) or a control rAAV‐CMV‐DIO‐mCherry vehicle (AAV‐mCherry). (B‐D) The expressions of circRNA_34414, miR‐6960‐5p and SIRT3 mRNA in CA1 region among the four groups. (B: *N* = 6, *F*(3,20) = 50.31, *p* < 0.01, One‐way ANOVA; C: *N* = 6, *F*(3,20) = 20.51, *p* < 0.01, One‐way ANOVA; D: *N* = 6, *F*(3,20) = 45.48, *p* < 0.01, One‐way ANOVA). (E) Protein levels of SIRT3 estimated by western blot among the four groups (*n* = 6, *F*(3,20) = 22.51, *p* < 0.01, One‐way ANOVA). (F) The percentage of co‐labeling number of SIRT3 and CaMKIIα+ in CA1 region among the four groups (*n* = 6, *F*(3,20) = 85.74, *p* < 0.01, One‐way ANOVA). (G) Representative images of CaMKIIα+ stained with SIRT3 (Scale bar: 100 μm) in hippocampal CA1 region of left. (H,I) Protein levels of SOD2 and AC‐SOD2 estimated by western blot among the four groups (H: *N* = 6, *F*(3,20) = 0.35, *p* = 0.79, One‐way ANOVA; I: *N* = 6, *F*(3,20) = 9.70, *p* < 0.01, One‐way ANOVA). (J) The levels of MMP in mitochondria among the four groups (*n* = 6, *F*(3,20) = 10.32, *p* < 0.01, One‐way ANOVA). (K) The latency to eat the food among the four groups (*n* = 10, *F*(3,36) = 10.55, *p* < 0.01, One‐way ANOVA). (L–N) The total distance, inner distance and inner time in OFT among the four groups (L: *N* = 10, *F*(3,36) = 1.44, *p* = 0.25, One‐way ANOVA; M: *N* = 10, *F*(3,36) = 11.25, *p* < 0.01, One‐way ANOVA; N: *N* = 10, *F*(3,36) = 7.85, *p* < 0.01, One‐way ANOVA). (O–Q) The total distance, novel distance and novel time in Y maze among the four groups (O: *N* = 10, *F*(3,36) = 2.05, *p* < 0.01, One‐way ANOVA; P: *N* = 10, *F*(3,36) = 8.14, *p* < 0.01, One‐way ANOVA; Q: *N* = 10, *F*(3,36) = 10.61, *p* < 0.01, One‐way ANOVA). The data are plotted as Mean ± SEM. *p < 0.05, **p < 0.01 compared with the C+ AAV‐mCherry group. ^#^ *p* < 0.05, ^##^ *p* < 0.01 compared with the A/S+ AAV‐mCherry group.

Next, we knocked down miR‐6960‐5p in CA1 region of Vglut1‐cre mice (Figure 4A), the injection of rAAV‐4XmiR‐6960‐5p not change circRNA_34414 level in C + 4XmiR‐6960‐5p group compared with C + AAV‐mCherry group (Figure 4B), resulting in a significant decrease in miR‐6960‐5p expression in A/S + 4XmiR‐6960‐5p group compared with A/S + AAV‐mCherry group (Figure 4C), and reversed the decrease in SIRT3 mRNA (Figure 4D) and protein (Figure 4E) in the A/S + 4XmiR‐6960‐5p group. Immunofluorescence results showed a significant increase of SIRT3 expression in CaMKII⍺ + neurons (Figure 4F,G). Knocking down miR‐6960‐5p also reversed the increase of AC‐SOD2 (Figure 4H,I) and the decrease in MMP (Figure 4J). At the same time, knocking down miR‐6960‐5p could also reverse delirium‐like behavior in mice caused by anesthesia/surgery. The latency to eat food in the buried food test was rescued (Figure 4K). In the open field test, the distance and time in the inner zone were significantly increased (Figure 4M,N). The distance and time spent in the novel arm was significantly increased in the Y maze (Figure 4P,Q). In addition, there was no significant difference in the total distance in the open field and Y maze test (Figure 4L,O).

### 3.8 The circRNA_34414/miR‐6960‐5p axis participates in postoperative delirium of older mice

**FIGURE 5:**
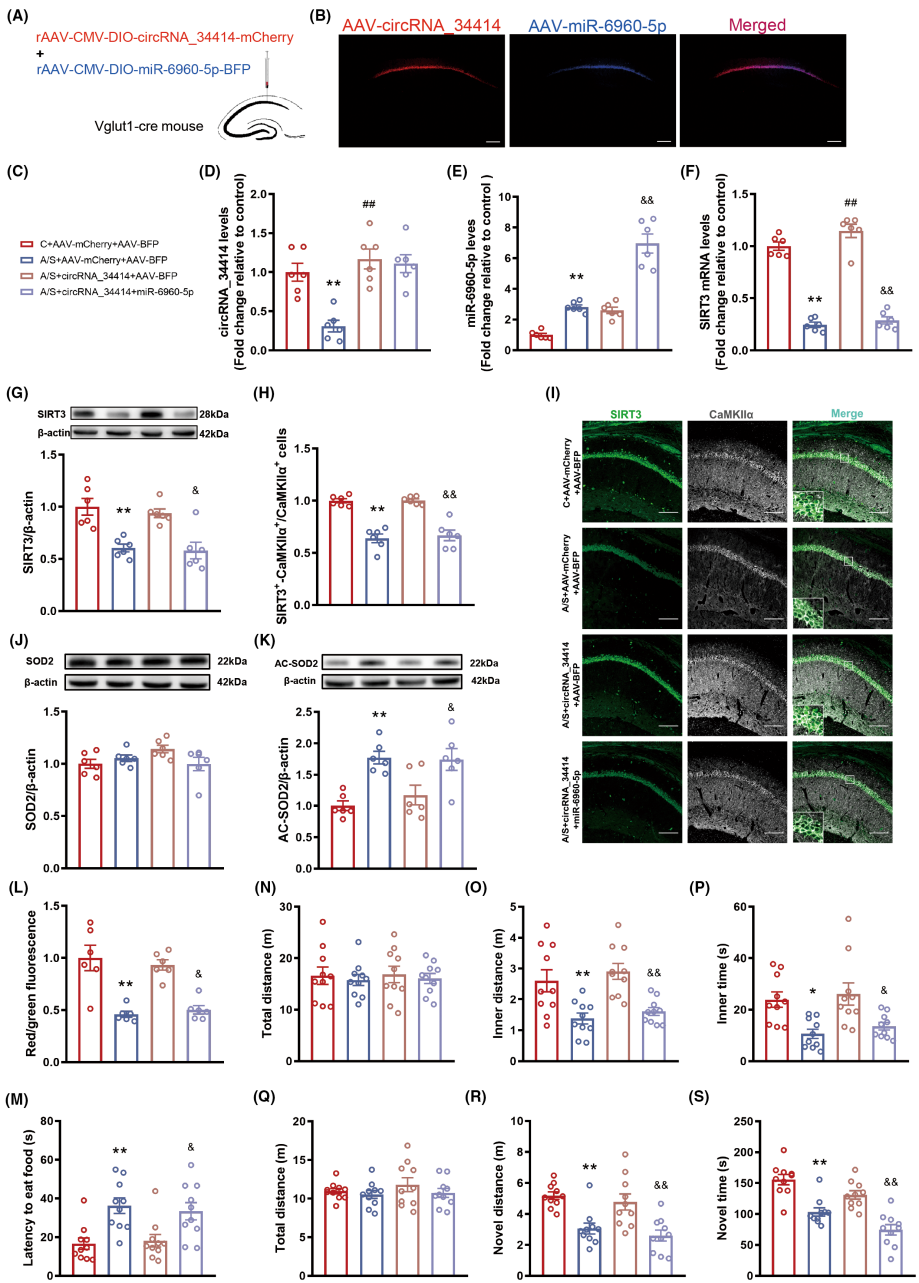
Effect of overexpression of circRNA_34414 and miR‐6960‐5p on postoperative delirium in Vglut1 mice. (A,B) The location and schematic for viral microinjection with a rAAV‐CMV‐DIO‐circRNA_34414‐mCherry or rAAV‐CMV‐DIO‐miR‐6960‐5p‐BFP. (C–F) The expressions of circRNA_34414, miR‐6960‐5p and SIRT3 mRNA in CA1 region among the four groups (D: *N* = 6, *F*(3,20) = 13.32, *p* < 0.01, One‐way ANOVA; E: *N* = 6, *F*(3,20) = 56.80, *p* < 0.0001, One‐way ANOVA; F: *N* = 6, *F*(3,20) = 118.6, *p* < 0.01, One‐way ANOVA). (G) Protein levels of SIRT3 estimated by western blot among the four groups (*n* = 6, *F*(3,20) = 12.18, *p* < 0.01, One‐way ANOVA). (H) The percentage of co‐labeling number of SIRT3 and CaMKIIα+ in CA1 region among the four groups (*n* = 6, *F*(3,20) = 33.07, *p* < 0.01, One‐way ANOVA). (I) Representative images of CaMKIIα+ stained with SIRT3 (Scale bar: 100 μm) in hippocampal CA1 region of left. (J–K) Protein levels of SOD2 and AC‐SOD2 estimated by western blot among the four groups (J: *N* = 6, *F*(3,20) = 2.21, *p* = 0.12, One‐way ANOVA; K: *N* = 6, *F*(3,20) = 8.84, *p* < 0.01, One‐way ANOVA). (L) The levels of MMP in mitochondria among the four groups (*n* = 6, *F*(3,20) = 16.14, *p* < 0.01, One‐way ANOVA). (M) The latency to eat food among the four groups (*n* = 10, *F*(3,36) = 7.44, *p* < 0.01, One‐way ANOVA). (N–P) The total distance, inner distance and inner time in OFT among the four groups (N: *N* = 10, *F*(3,36) = 0.27 *p* = 0.85, One‐way ANOVA; O: *N* = 10, *F*(3,36) = 9.28, *p* < 0.01, One‐way ANOVA; P: *N* = 10, *F*(3,36) = 7.09, *p* < 0.01, One‐way ANOVA). (Q–S) The total distance, novel distance and novel time in Y maze among the four groups (Q: *N* = 10, *F*(3,36) = 0.80, *p* = 0.50, One‐way ANOVA; R: *N* = 10, *F*(3,36) = 11.06, *p* < 0.01, One‐way ANOVA; S: *N* = 10, *F*(3,36) = 21.20, *p* < 0.01, One‐way ANOVA). The data are plotted as Mean ± SEM (*n* = 6). **p < 0.01 compared with the C + AAV‐mCherry + AAV‐BFP group. ^##^ *p* < 0.01 compared with the A/S + AAV‐mCherry + AAV‐BFP. ^&^ *p* < 0.05, ^&&^ *p* < 0.01 compared with the A/S + circRNA_34414 + AAV‐BFP.

The rAAV‐DIO‐circRNA_34414 overexpression and rAAV‐DIO‐miR‐6960‐5p overexpression mixed viruses or rAAV‐DIO‐circRNA_34414 and rAAV‐DIO‐BFP mixed viruses were laterally microinjected in the A/S group, whereas the rAAV‐DIO‐mCherry and rAAV‐DIO‐BFP viruses were microinjected in both the control and A/S groups of Vglut1‐cre mice (Figure 5A,B). This is the graphical representation of the groups (Figure 5C).

Injection of rAAV‐circRNA_34414 and rAAV‐miR‐6960‐5p did not change the circRNA_34414 expression (Figure 5D), but miR‐6960‐5p expression increased (Figure 5E). SIRT3 mRNA and protein levels decreased in the A/S + circRNA_34414 + miR‐6960‐5p group compared with those in the A/S + circRNA_34414 + AAV‐BFP group (Figure 5F,G), suggesting that the regulation of circRNA_34414 with SIRT3 was miRNA‐6960‐5p dependent. Immunofluorescence results showed a significant decrease of SIRT3 expression in CaMKII⍺ + neurons (Figure 5H,I). Overexpression of circRNA_34414 and upregulation of miR‐6960‐5p also countered the decrease of AC‐SOD2 (Figure 5J,K) and the increase of mitochondrial membrane potential (Figure 5L) in the hippocampal region of mice caused by circRNA_34414 overexpression. At the same time, it can also combat the effect of hippocampal circRNA_34414 overexpression on delirium‐like behavior in older mice. In the buried food test, the feeding latency of mice was significantly prolonged (Figure 5M). In the open field test, the distance and time in the inner zone were significantly reduced (Figure 5O,P). The distance and time spent in the novel arm in the Y maze test were also significantly reduced (Figure 5R,S). In addition, there was no significant difference in the total distance in the open field and the Y maze test (Figure 5N,Q).

### 3.9 The circRNA_34414/miR‐6960‐5p axis participates in postoperative delirium via SIRT3

**FIGURE 6:**
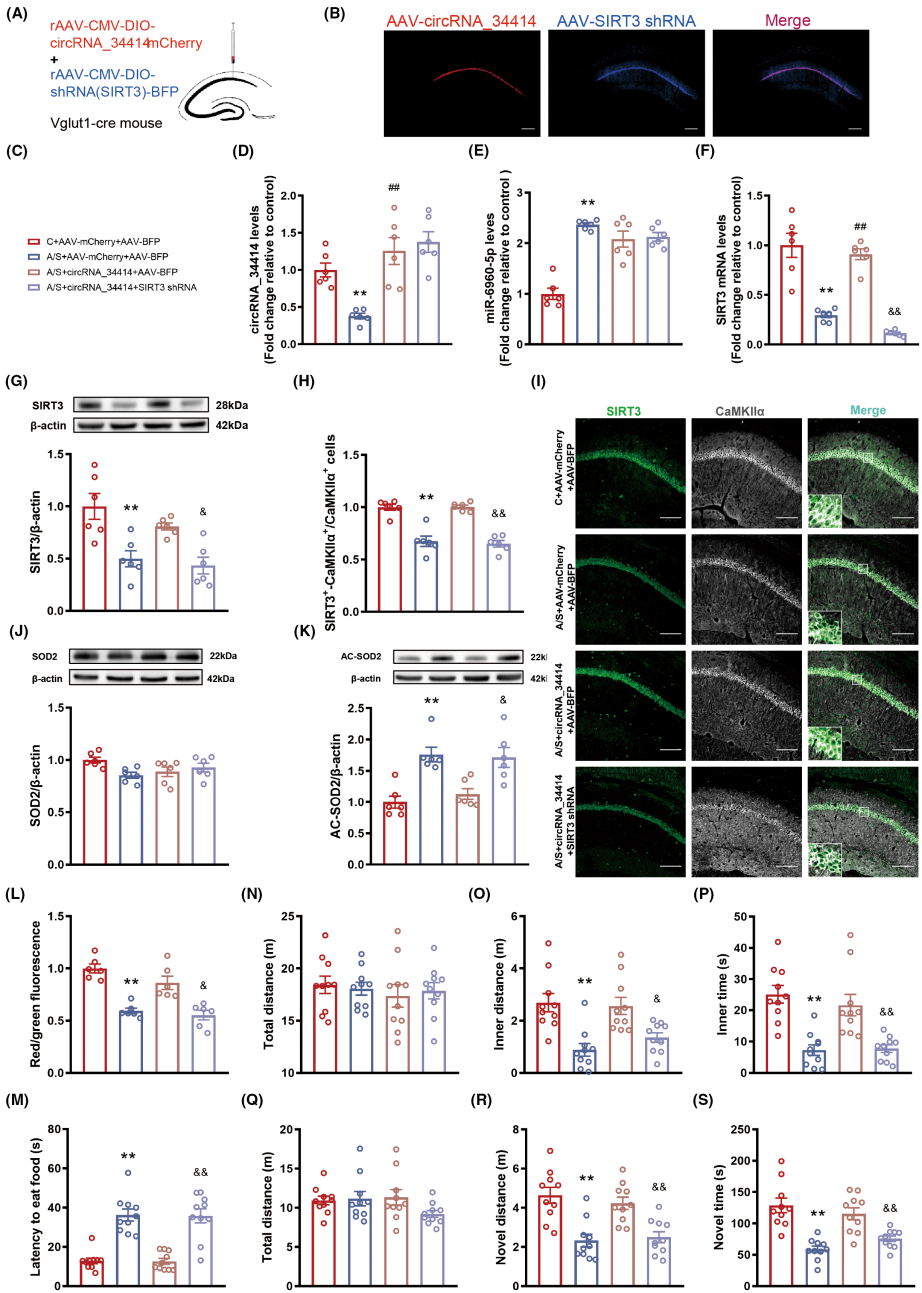
Effect of overexpression of circRNA_34414 and downregulation of SIRT3 on postoperative delirium in Vglut1 mice. (A,B) The location and schematic for viral microinjection with a rAAV‐CMV‐DIO‐circRNA_34414‐mCherry or rAAV‐CMV‐DIO‐shRNA(SIRT3)‐BFP. (C–F) The expressions of circRNA_34414, miR‐6960‐5p and SIRT3 mRNA in CA1 region among the four groups (D: *N* = 6, *F*(3,20) = 12.75, *p* < 0.01, One‐way ANOVA; E: *N* = 6, *F*(3,20) = 32.55, *p* < 0.01, One‐way ANOVA; F: *N* = 6, *F*(3,20) = 41.92, *p* < 0.01, One‐way ANOVA). (G) Protein levels of SIRT3 estimated by western blot among the four groups (*n* = 6, *F*(3,20) = 9.95, *p* < 0.01, One‐way ANOVA). (H) The percentage of co‐labeling number of SIRT3 and CaMKIIα+ in CA1 region among the four groups (*n* = 6, *F*(3,20) = 34.52, *p* < 0.01, One‐way ANOVA). (I) Representative images of CaMKIIα+ stained with SIRT3 (Scale bar: 100 μm) in hippocampal CA1 region of left. (J,K) Protein levels of SOD2 and AC‐SOD2 estimated by western blot among the four groups (J: *N* = 6, *F*(3,20) = 2.89, *p* = 0.06, One‐way ANOVA; K: *N* = 6, *F*(3,20) = 11.05, *p* < 0.01, One‐way ANOVA). (L) The levels of MMP in mitochondria among the four groups (*n* = 6, *F*(3,20) = 21.25, *p* < 0.01, One‐way ANOVA). (M) The latency to eat food among the four groups (*n* = 10, *F* (3,36) = 27.04, *p* < 0.01, One‐way ANOVA). (N–P) The total distance, inner time and inner distance in OFT among the four groups (N: *N* = 10, *F*(3,36) = 0.1282, *p* = 0.94, One‐way ANOVA; O: *N* = 10, *F*(3,36) = 9.94, *p* < 0.01, One‐way ANOVA; P: *N* = 10, *F*(3,36) = 14.22, *p* < 0.01, One‐way ANOVA). (Q–S) The total distance, novel time and novel distance in Y maze among the four groups (Q: *N* = 10, *F*(3,36) = 1.68, *p* = 0.19, One‐way ANOVA; R: *N* = 10, *F*(3,36) = 12.41, *p* < 0.01, One‐way ANOVA; S: *N* = 10, *F*(3,36) = 15.94, *p* < 0.01, One‐way ANOVA). The data are plotted as Mean ± SEM (*n* = 6). **p < 0.01 compared with the C+ AAV‐mCherry + AAV‐BFP group. ^##^ *p* < 0.01 compared with the A/S + AAV‐mCherry + AAV‐BFP. ^&^ *p* < 0.05, ^&&^ *p* < 0.01 compared with the A/S+ circRNA_34414 + AAV‐BFP.

The rAAV‐DIO‐circRNA_34414 overexpression and rAAV‐DIO‐SIRT3 knockdown shRNA viruses or rAAV‐DIO‐circRNA_34414 and rAAV‐DIO‐BFP viruses were laterally microinjected into Vglut1‐cre mice in the A/S group, whereas the rAAV‐DIO‐mCherry and rAAV‐DIO‐BFP viruses were microinjected into the Vglut1‐cre mice in both the control and A/S groups (Figure 6A,B). This is the graphical representation of the groups (Figure 6C).

Injection of rAAV‐circRNA_34414 and rAAV‐SIRT3 shRNA did not change the circRNA_34414 (Figure 6D) or miR‐6960‐5p expression (Figure 6E), SIRT3 mRNA (Figure 6F) and protein expression (Figure 6G) were significantly reduced, reversing the effect of overexpression circRNA_34414 increasing the expression of SIRT3 in glutamatergic neurons (Figure 6I,H). Overexpression of circRNA_34414 and downregulating SIRT3 also countered the decreasing in AC‐SOD2 with circRNA_34414 overexpression (Figure 6J,K) and the increase in MMP (Figure 6L). At the same time, it can also counter the effect of hippocampal circRNA_34414 overexpression on delirium‐like behavior in elderly mice. In the buried food test, the feeding latency of mice was significantly prolonged (Figure 6M). In the open field test, the distance and time in the inner zone was significantly reduced (Figure 6O,P). The distance and time spent in the novel arm in the Y maze test were significantly reduced (Figure 6R,S). In addition, there was no significant difference in the total distance in the open field and the Y maze test (Figure 6N,Q).

## 4 DISCUSSION

Postoperative delirium (POD) is one of the most common neurologic complications after anesthesia/surgery, primarily in the elderly population.^5^, ^24^, ^25^ Numerous clinical studies have shown that POD may lead to prolonged hospital stay, increased hospital costs, greatly increased patient burden, and affected postoperative recovery.^5^ Other studies have suggested that POD may lead to long‐term cognitive dysfunction and even symptoms such as dementia.^26^, ^27^

The main pathogenesis of postoperative delirium is not well understood, but recent studies have found that POD is associated with mitochondrial dysfunction, manifested by energy deficiency and overactivated oxidative stress.^28^, ^29^, ^30^ Mitochondria play a key role in the occurrence and development of neurodegenerative diseases.^31^ SIRT3 can regulate mitochondrial function and is associated with many aging‐related diseases such as cardiovascular disease and neurodegenerative disease.^8^ However, the role and mechanism of SIRT3 in the occurrence and development of POD are not well known.

In this study, we used behaviors including buried food test, open field test, and Y‐maze test to evaluate behavioral changes after surgery/anesthesia in older mice,^32^ and found that the surgical model of tibial fracture under sevoflurane anesthesia caused delirium‐like behavior. In the buried food test, the latency to eat food was significantly increased after anesthesia/surgery. The result shows that the motivation and ability of eating and the sense of smell of mice were damaged, which further indicated that the levels of attention, thinking and consciousness of mice were impaired. In OFT, We found that the anesthesia/surgery did not significantly change the total distance of the mice, indicating that the anesthesia/surgery did not impair the motor ability of the mice. The total distance of mice in Y maze also shows this problem. We also found that the anesthesia/surgery decreased the time and the distance spent in the center. Note that these behaviors also require the presence and intactness of attention, consciousness and organized thinking in the mice. In the Y‐maze test, the distance and the time spent in the novel arm was lower. Note that the hippocampus dependent spatial memory of mice was damaged, which also requires the presence and intactness of attention, consciousness and organized thinking. In conclusion, the anesthesia/surgery was able to impair certain natural and learned behaviors in mice that are dependent on attention, consciousness and organized thinking. We used electrophysiological, optogenetic and chemical genetic techniques to demonstrated that anesthesia/surgery reduced the excitability of glutamatergic neurons in CA1 region. In addition, we confirmed that anesthesia/surgery reduced SIRT3 expression and mitochondrial function. Furthermore, the decrease of SIRT3 was specific in glutamatergic neurons of the CA1 region. By cell‐type specific overexpression of SIRT3 could significantly protect postoperative delirium. In the meanwhile, SIRT3 overexpression releases the amelioration of anesthesia/surgery‐induced mitochondrial dysfunction.

**FIGURE 7:**
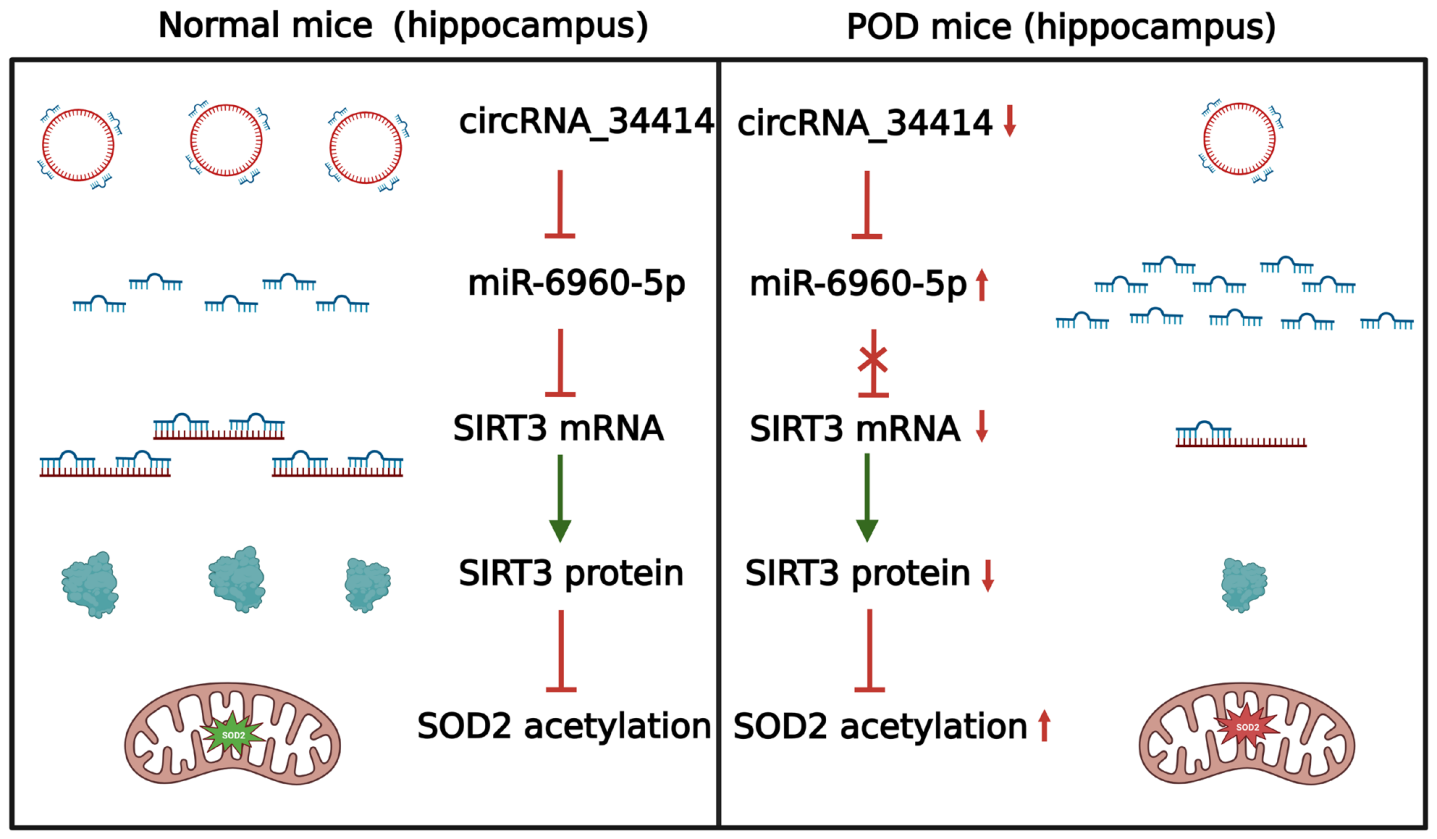
Schematic diagram depicting the circRNA_34414/miR‐6960‐5p/SIRT3 axis in postoperative delirium of older mice. After anesthesia/surgery, the reduction in circRNA_34414 levels leads to more miR‐6960‐5p, decreased SIRT3 mRNA, lower SIRT3 protein levels, increased the acetylation of SOD2, which ultimately results in mitochondrial dysfunction and postoperative delirium.

CircRNAs are evolutionarily conserved at the level of different species sequences and are extremely rich, conserved, and dynamically expressed in mammalian brains.^33^ Since CircRNA is preferentially expressed in neural genes and neural tissues, it is indicated that circRNAs are closely related to neurological diseases such as Alzheimer's disease, one of the most common neurodegenerative diseases.^34^, ^35^ CircRNA has been reported to have various regulatory effects, including interaction with RNA‐binding proteins, acting as miRNA sponges that regulate the transcription of paternal genes primarily at the post‐transcriptional and transcriptional levels. Approximately 70% of miRNAs have been found in the brain and neurons,^36^ meaning that miRNAs play an important regulatory function during the development of the nervous system. Deregulation of miRNAs involves neurodegenerative abnormalities such as Parkinson's disease (PD) and Alzheimer's disease (AD).^37^ We screened the miRNA bound to SIRT3 as miR‐6960‐5p through transcriptome sequencing, target gene prediction and GEO database, and further confirmed that the circRNA bound to miR‐6960‐5p was circRNA_34414. qRT‐PCR assay determined that circRNA_34414 and SIRT3 expression decreased and miR6960‐5p expression increased after anesthesia/surgery. SIRT3 interacted with miR6960‐5p and miR6960‐5p interacted with circRNA_34414 with diluciferase reporter gene experiments. In situ hybridization experiments and nucleoplasmic separation experiments confirmed that circRNA_34414 and miR6960‐5p were mainly located in the cytoplasm, suggesting that circRNA_34414 was involved in the ceRNA mechanism. Next, we found that overexpression of circRNA_34414 significantly improved the decline in SIRT3 expression and delirium‐like behavior in elderly mice caused by anesthesia/surgery. Knockdown miR‐6960‐5p was consistent with overexpression of circRNA_34414. The circRNA_34414 and miR‐6960‐5p overexpression or circRNA_34414 overexpression and SIRT3 downregulation mixed virus counteracted the effect of circRNA_34414 overexpression on postoperative delirium. The above results suggest that circRNA_34414 may regulate the expression of SIRT3 through the ceRNA mechanism by targeting miR‐6960‐5p, and this process may play an important role in the mechanism of postoperative delirium (Figure 7).

Although we confirmed that circRNA_34414 has miRNA sponge effect, whether it has other functions such as binding proteins and regulating the cleavage of certain genes is unknown, and further exploration is still needed. Other studies reported that the extracellular Alpha‐synuclein (ASN) or transcription factor NRF2 could also regulate the SIRT3 expression in Alzheimer's disease,^38^, ^39^ whether these were involved in the change of SIRT3 of postoperative delirium in mice need further investigation in the following studies. The role of SIRT3 in neurodegenerative diseases is not fully understood, while current research suggests that a decline in SIRT3 expression leads to hyperacetylation of hundreds of mitochondrial proteins, associated with abnormalities in the nervous system, death of nerve cells^40^; Silencing of SIRT3 also leads to oxidative damage to neurons.^41^ Although this project confirms the key role of SIRT3 in the occurrence and development of POD, its specific mechanism of action is still unclear and requires further in‐depth study. We have not ruled out whether other SIRTs family members are also involved in the formation of POD, and the interaction between various SIRTs family members and the differences in regulating the occurrence and development of POD need to be further elucidated.

In summary, our study found that circRNA_34414 can act as CERNA to block the inhibitory effect of miR‐6960‐5p on SIRT3, thereby participating in the occurrence of postoperative delirium. These data provide new insights into the pathogenesis of postoperative delirium and identify potential therapeutic targets.

## AUTHOR CONTRIBUTIONS

Supervision and funding acquisition: YQW, TZL, HHM. Conceptualization: HBW, QL, YPL, WD, JW, XHJ. Data curation and formal analysis: HBW, QL, YPL. Writing‐original draft: HBW, HHM. Writing‐ review & editing: TZL, YQW, HHM. Approval of final version of the manuscript: all authors.

## FUNDING INFORMATION

The Natural Science Foundation of Beijing (7212023), National Natural Science Foundation of China (82071180, 82271206, 82171191, 81971051, 82371211), the Key Subject of Colleges and Universities Natural Science Foundation of Jiangsu Province (23KJA320009), the Postgraduate Research & Practice Innovation Program of Jiangsu Province (KYCX22_2870).

## CONFLICT OF INTEREST STATEMENT

The authors declare that they have no conflicts of interest.

## Supporting information


Data S1

Figure S1

Figure S2

Figure S3

Figure S4

Figure S5

Files S1

## Data Availability

The data that support the findings of this study are available from the corresponding author upon reasonable request.
