# Supplementary material for: Role of the circRNA_34414/miR‐6960a‐5p/SIRT3 axis in postoperative delirium via CA1 Vglut1+ neurons in older mice
Source: CNS Neurosci Ther. 2024 Aug 13;30(8):e14902. doi: 10.1111/cns.14902 (PMC11322041; doi:10.1111/cns.14902)
Supplement: Supplementary file 1 — Data S1 [file CNS-30-e14902-s006.docx]

**Role of the circRNA_34414/miR-6960a-5p/SIRT3 Axis in Postoperative Delirium via CA1 Vglut1+ Neurons in Older Mice**

Hai-Bi Wang^1,a^, Qiang Liu^1,a^, Yan-Ping Liu ^1,a^, Wei Dong^1^, Jie Wan^1^, Xin-Hao Jiao^1^, Yu-Qing Wu^1,^*,Tian-Zuo Li^2,^*, Hui-Hui Miao^2,^*

# 1 MATERIALS AND METHODS

**1.1 POD mouse model**

POD models were created using the intramedullary fixation technique for tibial fractures[1]. Make a 1 cm incision longitudinally under the knee of the mouse's left hind paw, separate the muscle, expose the tibia, insert a 0.4 mm stainless steel needle at the tibial tuberosity into the lumens of the mouse tibial bone marrow, clamp it in the middle of the tibia with surgical forceps, and then suture the incision and the muscle. Before cutting the skin, 2% lidocaine is injected subcutaneously, and all experimental procedures rigorously adhere to the sterility principle. For the treatment, the mice in the control group (C) received none anesthesia/surgery procedure; in the anesthesia/surgery group (A/S), the mice received 2.0% isoflurane for maintenance with surgery.

**1.2 Buried food test**

Consistent with previous studies [2-4]. The mice were allowed to explore the oat pellets that were hidden 0.5 cm deep beneath the litter. The time taken by the mice to find the food was recorded. If the mice were unable to locate sustenance within 5 minutes, the duration was documented as 300 seconds. After each test, the test cage was clean with 70% ethanol solution and subsequently replace it with fresh litter.

**1.3 Open field test**

Following previous studies[4, 5], mice were placed into a test box measuring 50 cm x 50 cm x 50 cm. They were allowed to freely explore the environment for a duration of 5 minutes. The overall distance and the time/distance within the inner zone were record. At the end of each test, we wipe the test cage with a 70% ethanol solution.

**1.4 Y maze test**

During the adaption phase, one arm of the Y maze is closed. Mice are first placed in the starting arm, then allowed to explore freely for 10 minutes while the distance and time of their movement in each arm were recorded. After two hours, the testing phase begins, and the new arm were opened. The mice explored for 5 minutes, and the distance and time spent in the novel arm were record[4, 6]. At the end of each excursion, wipe each arm with 70% alcohol.

**1.5 Western Blotting**

The main antibodies we employed were as follows: SIRT3 (1:1000, 5490, Cell Signaling Technologies, USA), SOD2 (1:1000, 13141, Cell Signaling Technologies, USA), and ac-SOD2 (acetyl K68) (1:1000, ab137037, Abcam, UK). The color was produced using ultrasensitive ECL chemiluminescence solution after the secondary antibody was applied, and the image was analyzed using Image J software.

**1.6 Determination of mitochondrial membrane potential (MMP)**

After extracting mitochondria with the tissue mitochondrial isolation kit[7], the mitochondrial membrane potential was detected by JC-1 method, and the diluted JC-1 staining solution was prepared according to the instructions. Take 0.9 ml of the diluted JC-1 staining solution and add 0.1ml of purified mitochondria. The JC-1 monomer was identified first, with the excitation light set to 490 nm and the emitted light set to 530 nm; the JC-1 polymer was then detected, with the excitation light set to 525 nm and the emitted light set to 590 nm.

**1.7 Immunofluorescence**

Mice were perfused with 4% paraformaldehyde and taken from the brain and dehydrated with a gradient of 20% and 30% sugar water for three days until the tissue was submerged. Cut the brain tissue of the sugar sink to a thickness of 25um. Incubate the primary antibody after blocking with 5% goat serum for 1 h and incubate at 4 °C for 48 h. We used the following primary antibodies for immunofluorescence: anti-SIRT3 (1:100, orb103490 Biorbyt) mixed with anti-GAD67 (1:300, ab26116, Abcam) and anti-SIRT3 mixed with anti-CamKIIα (1:300, 50049S, Cell Signaling Technology). Remove the brain slice and immerse in the following secondary antibody incubation solution: goat anti-rabbit IgG (bound to 488 nm fluorescence, 1:500), goat anti-mouse IgG (bound to 594 nm fluorescence, 1:500), incubate at 37 °C for 2 h. Among them, the primary antibody and secondary antibody dilutions were both 0.3% PBST. In a light-protected environment, the brain film is attached to a glass slide and allowed to air dry. Subsequently, the DAPI-containing tablet is administered in a dropwise manner. Fluorescence pictures were acquired with laser confocal microscopy and afterwards subjected to statistical analysis utilising the Image J software.

**1.8 RNA antisense purification assays (RAP)**

A mmu_circRNA_34414 biotinylated probe (5Biotin- ATAAAGTGCCTTAACCTT CCAATTTAACACCA-3′) was designed and synthesized by BersinBio Technologies (Guangzhou, China). Biotin-labeled RNA pull-down was performed using an RNA Antisense Purification Kit (Bes5103-1, BersinBio, China) according to the manufacturer’s protocol. The enriched RNA was detected using qRT-PCR.

**1.9 qRT-PCR**

In accordance with earlier research[8], Extraction of hippocampal tissue total RNA with Trizol kit (Invitgen, Carlsad, CA) and transcription into cDNA with reverse transcription kit (R223 Vazyme Nanjing). The MiRNA Extraction and Reverse Transcription MiRNA Rapid Extraction Kit and the MiRNA Reverse Transcription Kit (R015-50 MR101) were performed for RT-qPCR analysis on the Roche 480 Light Cycler detection system. Each sample was analyzed in duplicate. The primer sequences used are shown in Supplementary Table 1.

**1.10 Adeno-Associated Virus (AAV) Vectors and Viral Injections**

rAAV-CMV-DIO-SIRT3-P2A-mCherry-WPRE-hGHpolyA, (AAV-SIRT3) vector and a control vector rAAV-CMV-DIO- mCherry-WPRE-hGHpolyA, (AAV-mCherry).A rAAV-CMV- DIO-circRNA_34414-mCherry-WPRE-hGHpolyA,(AAV-circRNA_34414) vector and a control vector rAAV-CMV-DIO-mCherry-WPRE-hGHpolyA, (AAV-mCherry), and a rAAV-CMV-DIO-4XmiR-6960-5p-mCherry-WPRE-hGHpolyA, (AAV-4XmiR-6960-5p) vector and a control vector rAAV-CMV-DIO-mCherry-WPRE-hGHpolyA, (AAV-mCherry) . rAAV-CMV-DIO-miR-6960-5p-BFP-WPRE-hGHpolyA,(AAV-miR-6960-5p) vector and a control vector rAAV-CMV-DIO-BFP-WPRE-hGHpolyA,(AAV-BFP), and a rAAV-CMV-DIO-shRNA(SIRT3)-BFP-WPRE-hGHpolyA,(AAV-SIRT3 shRNA) and a control vector rAAV-CMV-DIO-BFP-WPRE-hGHpolyA,(AAV-BFP). All the above adeno-associated viruses were prepared by Wuhan Shunmi Technology Co., Ltd. A volume of 0.15-0.5 ml virus was delivered bilaterally into the hippocampal CA1 of mice (anteroposterior, -2.0 mm; mediolateral, ± 1.5 mm; dorsoventral, -1.4 mm) at a rate of 0.1 ml/min. Viral injection sites were histologically confirmed by the fluorescence signal in the hippocampal CA1 of brain slices using a fluorescence microscope.

**1.11 Subcellular Localization Measurement**

Subcellular localization of circRNA_34414 and miR-6960-5p was determined by fluorescence in situ hybridization (FISH) and nuclear and cytoplasmic fractionation. For FISH analysis, HT22 cells grown on the slides were fixed in 4% paraformaldehyde for 20 minutes, and then treated with protease K. After incubation with prehybridization buffer at 37°C for 1 hour, the cells were hybridized with a carboxy fluorescein (FAM)-labeled specific probe targeting to circRNA_34414 and miR-6960-5p (Han Heng Biotechnology Co., Ltd., Shang Hai, China) at 37°C overnight. Then, cell nuclei were counterstained with DAPI (4’,6-diamidino-2-phenylindole). For nuclear and cytoplasmic fractionation analysis, nuclear and cytosolic fractions were separated using a Cytoplasmic & Nuclear RNA Purification Kit (Norgenbiotek, Canada) according to the manufacturer’s instruction. The expression level of circRNA_34414 in the nuclear and cytoplasm was detected by RT-qPCR assays. GAPDH used as control.

**1.12 Dual-Luciferase Reporter Assay**

The luciferase reporter vectors containing the predicted binding sequence of miR-6960-5p in circRNA_34414 (mmu-circRNA_34414-wt) or SIRT3-3UTR (m-SIRT3-3UTR-wt) and their mutated sequence (mmu-circRNA_34414-mut or m-SIRT3-3UTR-mut) were constructed by Han Heng bio Co., Ltd. (Shanghai, China). HT22 cells were cultured to approximately 70% confluence, then the mmu-circRNA_34414-wt or mmu-circRNA_34414-mut vectors were transfected into HT22 cells with miR-6960-5p mimics or miR-6960-5p NC. Similarly, the m-SIRT3-3UTR -wt or m-SIRT3-3UTR -mut vectors were transfected into HT 22 cells with miR-6960-5p mimics or miR-6960-5p NC. After 48-hour, the cells were harvested, and Firefly and Renilla luciferase activities were measured using a dual-luciferase reporter system (Promega, USA) according to the manufacturer’s instructions.

**1.13 In vitro brain slice electrophysiology**

Mice were anesthetized with sevoflurane and were intracardially perfused with 20 mL oxygenated ice-cold high sucrose solution that contained (in mM): 80 NaCl，3.5KCl，4.5 MgSO4，0.5 CaCl2，1.25 NaH2PO4·2H2O，90 Sucrose，25 NaHCO3，10 Glucose. Acute brain slices (300 um) containing Hippocampus were sectioned in chilled (2‒4℃) high sucrose solution on a microtome (VT1200s, Leica, Germany) vibrating at 0.12 mm/s velocity[9]. The brain slices were initially incubated in high sucrose solution (saturated with 95% O2/5% CO2 to provide stable pH and continuous oxygenation) which was followed by recovery for at least 1 hour. Then the brain slices were transferred to a slice chamber for electrophysiological recording with continuous perfusion with standard ACSF that contained (in mM): 126 NaCl, 2.5 KCl, 1.2 NaH2PO4, 1.2 MgSO4·7H2O, 2.4 CaCl2·2H2O, 26 NaHCO3, 10 Glucose. (pH: 7.3‒7.4, osmolarity: 300‒310 mOsm).

Whole-cell patch-clamp recordings were performed using patch pipettes (5‒8 MΩ). The pipettes contained (in mM): 10 Phosphocreatine-Tris, 10 HEPES, 0.3 EGTA, 2 ATP-Mg, 0.5 GTP-Na, 115 K-Gluconate, 20 KCl, 1.5 MgCl2, pH 7.2, 280-290 mOsm. The signals were collected using a MultiClamp 700B amplifier, low-pass-filtered at 2 kHz and digitized at 10 kHz with a Digidata 1440B Data Acquisition System and analyzed using the software pClamp10.7 (Molecular Devices, Sunnyvale, USA). Sixteen neurons were counted for statistical analysis. Specifically, four animals in each group were utilized, and four neurons in the CA1 region of each coronal slice of a mouse were selected for analysis.

**1.14 Optogenetics**

AAV-CaMKIIa-eNpHR-mCherry or AAV-CaMKIIa-hChR2-EGFP positive neurons were selected, and the action potential release changes before and after continuous yellow light irradiation (589nm, 0.001Hz) or blue light irradiation (470nm, 20Hz) were recorded after applying 30pA depolarization current induced neurons under current clamp. After 3 weeks of virus injection and fiber insertion, behavioral testing under optogenetic regulation was performed. Before the behavioral test, the fiber mortise pin above the mouse hippocampus CA1 was connected to the laser light source through the fiber jumper, and the illumination mode was set to yellow, 589nm, 0.001Hz, 4mW, duration 8s, interval 2s. Blue light, 470nm, 20 Hz, 15ms, 4mW.

**1.15 Chemical genetics**

AAV-EF1α-DIO-hM4Di-mCherry or AAV-EF1α-DIO-hM3Dq-mCherry-positive neurons were selected and electrophysiological recordings were performed, and the frequency of action potential emission of cells under 150pA current stimulation before and after CNO (10μM) perfusion solution was recorded.

Supplementary Table 1. The primer sequences.

| Gene name | Primer sequence |
| --- | --- |
| circRNA_34414 | F:GACAGCATCAGCTCAGAGGTT |
|  | R:GGATAAGGTCTCCCTCGGGTT |
| SIRT3 | F:AGTGACATTGGGCCTGTAGTG |
|  | R:TACCCTGAAGCCATCTTTGAA |
| miR-6960-5p | F:CGCGCAGGATGAGAAGAGTT |
|  | R:AGTGCAGGGTCCGAGGTATT |
|  | RT:GTCGTATCCAGTGCAGGGTCCGAGG TATTCGCACTGGATACGACCAGCCA |
| ACTB | F:GGCTGTATTCCCCTCCATCG |
|  | R: CCAGTTGGTAACAATGCCATGT |
| U6 | F:AGAGAAGATTAGCATGGCCCCTG |
|  | R:ATCCAGTGCAGGGTCCGAGG |
|  | RT:GTCGTATCCAGTGCAGGGTCCGAGGTATTCGCACTGGATACGACAAAATA |

# 2 Figure legends

Fig S1. Anesthesia and surgery reduced Vglut1+ neuron activation in the CA1 region.

(A) Whole-cell path recording picture. (B) Threshold currents of the action potential (C group: *n*=16 neurons, A/S group: *n*=15 neurons; *P*<0.01, C vs A/S, t-test.). (C)Schematic illustration of action potentials evoked by depolarizing current. (D) Firing rate of action potentials evoked by depolarizing current pulses of 0–150 pA. (C group: *n*=16 neurons, A/S group: *n*=15 neurons; *P*<0.01, C vs A/S, two-way ANOVA.). (E) Comparison of resting membrane potential. (C group: *n*=16 neurons, A/S group: *n*=15 neurons; *P*=0.62, C vs A/S, t-test.).(F) Changes in action potential under blue light irradiation. (G) Changes of action potential under yellow light irradiation. (H) Schematic diagram of virus injection and fiber placement in CA1 region of Vglut1-cre mice. (I) The latency to eat food in buried food test at postoperative 6 hours after blue light irradiation in the A/S group mice. (*n*=10, *P*<0.01, AAV-eGFP vs AAV-ChR2, t-test.). The total distance (J: *n*=10, *P*=0.34, AAV-eGFP vs AAV-ChR2, t-test.), the inner distance (K: *n*=10, *P*<0.01, AAV-eGFP vs AAV-ChR2, t-test.) and the inner time (L: *n*=10, *P*<0.01, AAV-eGFP vs AAV-ChR2, t-test.) in the open field test at postoperative 6 hours after blue light irradiation in the A/S group mice. The total distance (M: *n*=10, *P*=0.92, AAV-eGFP vs AAV-ChR2, t-test.), the novel distance (N: *n*=10, *P*<0.01, AAV-eGFP vs AAV-ChR2, t-test.) and the novel time (O: *n*=10, *P*<0.01, AAV-eGFP vs AAV-ChR2, t-test.) in the Y maze test at postoperative 6 hours after blue light irradiation in the A/S group mice. (P) Schematic showing AAV-DIO-ChR2-eGFP virus in CA1 region 3 weeks after injection. (Q) The latency to eat food in buried food test in control mice after yellow light irradiation (*n*=10, *P*<0.01, AAV-mCherry vs AAV-eNpHR, t-test.). The total distance (R: *n*=10, *P*=0.14, AAV-mCherry vs AAV-eNpHR, t-test.), the inner distance (S: *n*=10, *P*<0.01, AAV-mCherry vs AAV-eNpHR, t-test.) and the inner time (T: *n*=10, *P*<0.01, AAV-mCherry vs AAV-eNpHR, t-test.) in the open field test in the control mice after yellow light irradiation. The total distance (U: *n*=10, *P*=0.44, AAV-mCherry vs AAV-eNpHR, t-test.), the novel distance (V: *n*=10, *P*<0.01, AAV-mCherry vs AAV-eNpHR, t-test.) and the novel time (W: *n*=10, *P*<0.01, AAV-mCherry vs AAV-eNpHR, t-test.) in the Y maze test in the control mice after yellow light irradiation. The data are plotted as Mean ± SEM. **p* <0.05, ***p* <0.01 compared with the control group.

Fig S2. Chemical genetics validate that anesthesia and surgery reduce glutamatergic neuron excitability in the CA1 region.

(A) The number of c-Fos/hM3Dq cells in CA1 at postoperative 6 hours. (B) Representative recordings showing evoked (80pA) action potentials in Vglut1 mice injected with hM3Dq-Saline or hM3Dq-CNO. (C) The latency to eat food in buried food test after CNO treatment in the A/S group Vglut1 mice (*n*=10, *P*<0.01, hM3Dq-Saline vs hM3Dq-CNO, t-test.). The total distance (D: *n*=10, *P*=0.27, hM3Dq-Saline vs hM3Dq-CNO, t-test.), the inner distance (E: *n*=10, *P*<0.01, hM3Dq-Saline vs hM3Dq-CNO, t-test.) and the inner time (F: *n*=10, *P*<0.01, hM3Dq-Saline vs hM3Dq-CNO, t-test.) in the open field test after CNO treatment in the A/S group Vglut1 mice. The total distance (G: *n*=10, *P*=0.55, hM3Dq-Saline vs hM3Dq-CNO, t-test.), the novel distance (H: *n*=10, *P*<0.01, hM3Dq-Saline vs hM3Dq-CNO, t-test.) and the novel time (I: *n*=10, *P*<0.01, hM3Dq-Saline vs hM3Dq-CNO, t-test.) in the Y maze test after CNO treatment in the A/S group Vglut1 mice. (J) The number of c-Fos/Hm4Di cells in CA1 region in the control group Vglut1 mice. (K) Representative recordings showing evoked (150pA) action potentials in Vglut1 mice injected with Hm4Di-Saline or hM4Di-CNO in CA1 region. (L) The latency to eat food in buried food test in the control group Vglut1 mice (*n*=10, *P*<0.01, hM4Di-Saline vs hM4Di-CNO, t-test.). The total distance (M: *n*=10, *P*=0.22, hM4Di-Saline vs hM4Di-CNO, t-test.), the inner distance (N: *n*=10, *P*<0.01, hM4Di-Saline vs hM4Di-CNO, t-test.) and the inner time (O: *n*=10, *P*<0.01, hM4Di-Saline vs hM4Di-CNO, t-test.) in the open field test in the control group Vglut1 mice. The total distance (P: *n*=10, *P*=0.13, hM4Di-Saline vs hM4Di-CNO, t-test.), the novel distance (Q: *n*=10, *P*=0.02, hM4Di-Saline vs hM4Di-CNO, t-test.) and the novel time (R: *n*=10, *P*=0.012, hM4Di-Saline vs hM4Di-CNO, t-test.) in the Y maze test in the control group Vglut1 mice. The data are plotted as Mean ± SEM. **p* <0.05, ***p* <0.01 compared with the control group.

Fig S3. **Anesthesia/surgery decreased the SIRT3 expressions and impaired the Mitochondrial function in CA1 region.**

(A-C) SIRT3 protein levels were estimated by Western Blot at postoperative 6, 9, and 24 hours. (A,6h: *n*=6, *P*<0.01, C vs A/S, t-test; B,9h: *n*=6, *P*<0.01, C vs A/S, t-test; C,24h: *n*=6, *P*=0.02, C vs A/S, t-test.). (D-F) SOD2 protein levels were estimated by Western Blot at postoperative 6, 9, and 24 hours. (D,6h: *n*=6, *P*=0.33, C vs A/S, t-test; E,9h: *n*=6, *P*=0.46, C vs A/S, t-test; F,24h: *n*=6, *P*=0.78, C vs A/S, t-test.). (G-I) AC-SOD2 protein levels were estimated by Western Blot at postoperative 6, 9, and 24 hours. (G,6h: *n*=6, *P*<0.01, C vs A/S, t-test; H,9h: *n*=6, *P*<0.01, C vs A/S, t-test; I,24h: *n*=6, *P*=0.03, C vs A/S, t-test.). (J-L) The levels of MMP in CA1 mitochondria of aged mice at postoperative 6, 9, and 24 hours. (J,6h: *n*=6, *P*<0.01, C vs A/S, t-test; K,9h: *n*=6, *P*<0.01, C vs A/S, t-test; L,24h: *n*=6, *P*=0.03, C vs A/S, t-test.). The data are plotted as Mean ± SEM. **p* <0.05, ***p* <0.01 compared with the control group.

**Fig S4.** Vglut1-specific **overexpression of SIRT3 improved the delirium-like behaviors.**

(A)Representative images of CaMKIIα+ and SIRT3 staining (Scale bar: 100 μm) in hippocampal CA1 region of left. (B) The percentage of SIRT3 and CaMKIIα^+^ co-labeling cells in hippocampal CA1 region (*n*=6, *P*<0.01, C vs A/S, t-test). (C)Representative images of GAD67^+^ and SIRT3 staining (Scale bar: 100 μm) in hippocampal CA1 region of left. (D) The percentage of SIRT3 and GAD67^+^ co-labeling cells in hippocampal CA1 region (*n*=6, *P*=0.57, C vs A/S, t-test). (E) Representative recordings showing evoked (150pA) action potentials in Vglut1 mice injected with AAV-SIRT3 in CA1 region. (F)Threshold currents that evoked the first action potential (*n*=16 neurons, F (3,44) =67.51, *P*<0.01, One-way ANOVA). (G) Firing rate of action potentials evoked by depolarizing current pulses of 0–150 pA (*n*=16 neurons; among groups, F(45,704)=18.66, *P*<0.01; currents, F(15,704)=277.2, *P*<0.001; group×currents, F(3,704)=906.3, *P*<0.01;Two-way ANOVA). (H) Comparison of resting membrane potential (*n*=16 neurons, F (3,44) =2.35, *P*=0.09, One-way ANOVA). (I) The latency to eat food in buried food test at postoperative 6 hours. (*n*=10, F (3,36) =6.08, *P*<0.01, One-way ANOVA). (J) The sample trace of open field test. (K) The total distance (*n*=10, F (3,36) =0.48, *P*=0.70, One-way ANOVA). (L) The inner distance (*n*=10, F (3,36) =13.67, *P*<0.01, One-way ANOVA) and the inner time (M: *n*=10, F (3,36) =9.58, *P*<0.01, One-way ANOVA) in the open field test at postoperative 6 hours. (N) The sample trace of Y maze test. The total distance (O: *n*=10, F (3,36) =0.99, *P*=0.41, One-way ANOVA), the novel distance (P: *n*=10, F (3,36) =5.76, *P*<0.01, One-way ANOVA) and the novel time (Q: *n*=10, F (3,36) =6.83, *P*<0.01, One-way ANOVA) in the Y maze test at postoperative 6 hours. The data are plotted as Mean ± SEM. **p* <0.05 ** *p* <0.01 compared with the C+VEH group. ^#^ *p* <0.05, ^##^ *p* <0.01 compared with the A/S+VEH group.

**Fig S5. Bioinformatic prediction of circRNA_34414/miR-6960-5p/SIRT3 axis.**

(A) Predicted circRNA_34414 binding site to miR-6960-5p. (B) Predicted miR-6960-5p binding site to SIRT3. (C-E) The expression level of circRNA_34414, miR-6960-5p and SIRT3 mRNA were estimated by qRT-PCR. (C: *n*=6, *P*<0.01, C vs A/S, t-test; D: *n*=6, *P*<0.01, C vs A/S, t-test; E: *n*=6, *P*<0.01, C vs A/S, t-test). ***p* <0.01 compared with the control group. (F) Schematic diagram of mmu-miR-6960-5p binding to the mmu-circRNA_34414 target site.(G) Mmu-miR-6960-5p interaction with mmu-circRNA_34414 by the diluciferase reporter. (*n*=3, F (3,8) =300.7, *P*<0.01, One-way ANOVA). ***p* <0.01 compared with the NC mimics+mmu_circRNA_34414-wt.(H) Schematic diagram of mmu-miR-6960-5p binding to the m-Sirt3-3UTR target site. (I) Mmu-miR-6960-5p interaction with m-Sirt3-3UTR by the diluciferase reporter (*n*=3, F (3,8) =509.7, *P*<0.01, One-way ANOVA). ***p* <0.01 compared with the NC mimics+m-Sirt3-3UTR-wt. (J) Nuclear slurry separation experiment and fluorescence in situ hybridization confirm the co-location of circRNA_34414 (green) and miR-6960-5p (red) in cytoplasm. Cell nucleuses were counterstained with DAPI (blue). (K-L) FISH test in HT22 cells showed the location of circRNA_34414 and miR-6960-5p in the cytoplasm. (*n*=3, *P*<0.01, NC probe vs circRNA_34414 probe, t-test). ***p* <0.01 compared with NC probe group. (M) RAP assay for miR-6960-5p level with circRNA_34414 probe input. (*n*=3, *P*=0.02, RAP vs NC, t-test). **P* <0.01 compared with RAP group. (N) Expression level of miR-6960-5p after upregulation of circRNA_34414. (*n*=6, *P*=0.62, Vector vs circRNA_34414, t-test). (O) Expression level of circRNA_34414 after upregulation of miR-6960-5p. (*n*=6, *P*=0.59, Vector vs miR-6960-5p, t-test). (P) Expression level of circRNA_34414 after downregulation of 4✕miR-6960-5p. (*n*=6, *P*=0.77, Vector vs 4XmiR-6960-5p, t-test). The data are plotted as Mean ± SEM.

# References

1. Velagapudi, R., et al., *Orthopedic Surgery Triggers Attention Deficits in a Delirium-Like Mouse Model.* Front Immunol, 2019. **10**: p. 2675.

2. Lu, Y., et al., *Surgery/Anesthesia disturbs mitochondrial fission/fusion dynamics in the brain of aged mice with postoperative delirium.* Aging (Albany NY), 2020. **12**(1): p. 844-865.

3. Rattazzi, L., et al., *Impaired sense of smell and altered olfactory system in RAG-1(-∕-) immunodeficient mice.* Front Neurosci, 2015. **9**: p. 318.

4. Peng, M., et al., *Battery of behavioral tests in mice to study postoperative delirium.* Sci Rep, 2016. **6**: p. 29874.

5. Liu, Q., et al., *Sirtuin 3 protects against anesthesia/surgery-induced cognitive decline in aged mice by suppressing hippocampal neuroinflammation.* Journal of Neuroinflammation, 2021. **18**(1).

6. Kraeuter, A.K., P.C. Guest, and Z. Sarnyai, *The Y-Maze for Assessment of Spatial Working and Reference Memory in Mice.* Methods Mol Biol, 2019. **1916**: p. 105-111.

7. Wolken, G.G. and E.A. Arriaga, *Simultaneous Measurement of Individual Mitochondrial Membrane Potential and Electrophoretic Mobility by Capillary Electrophoresis.* Analytical Chemistry, 2014. **86**(9): p. 4217-4226.

8. Ding, X., et al., *LncRNA XR_351665 Contributes to Chronic Pain-Induced Depression by Upregulating DNMT1 via Sponging miR-152-3p.* The Journal of Pain, 2023. **24**(3): p. 449-462.

9. Besing, G.K., et al., *Artificial sleep-like up/down-states induce synaptic plasticity in cortical neurons from mouse brain slices.* Front Cell Neurosci, 2022. **16**: p. 948327.
