# Supplementary figures and images for: Role of the circRNA_34414/miR‐6960a‐5p/SIRT3 axis in postoperative delirium via CA1 Vglut1+ neurons in older mice
Source: CNS Neurosci Ther. 2024 Aug 13;30(8):e14902. doi: 10.1111/cns.14902 (PMC11322041; doi:10.1111/cns.14902)

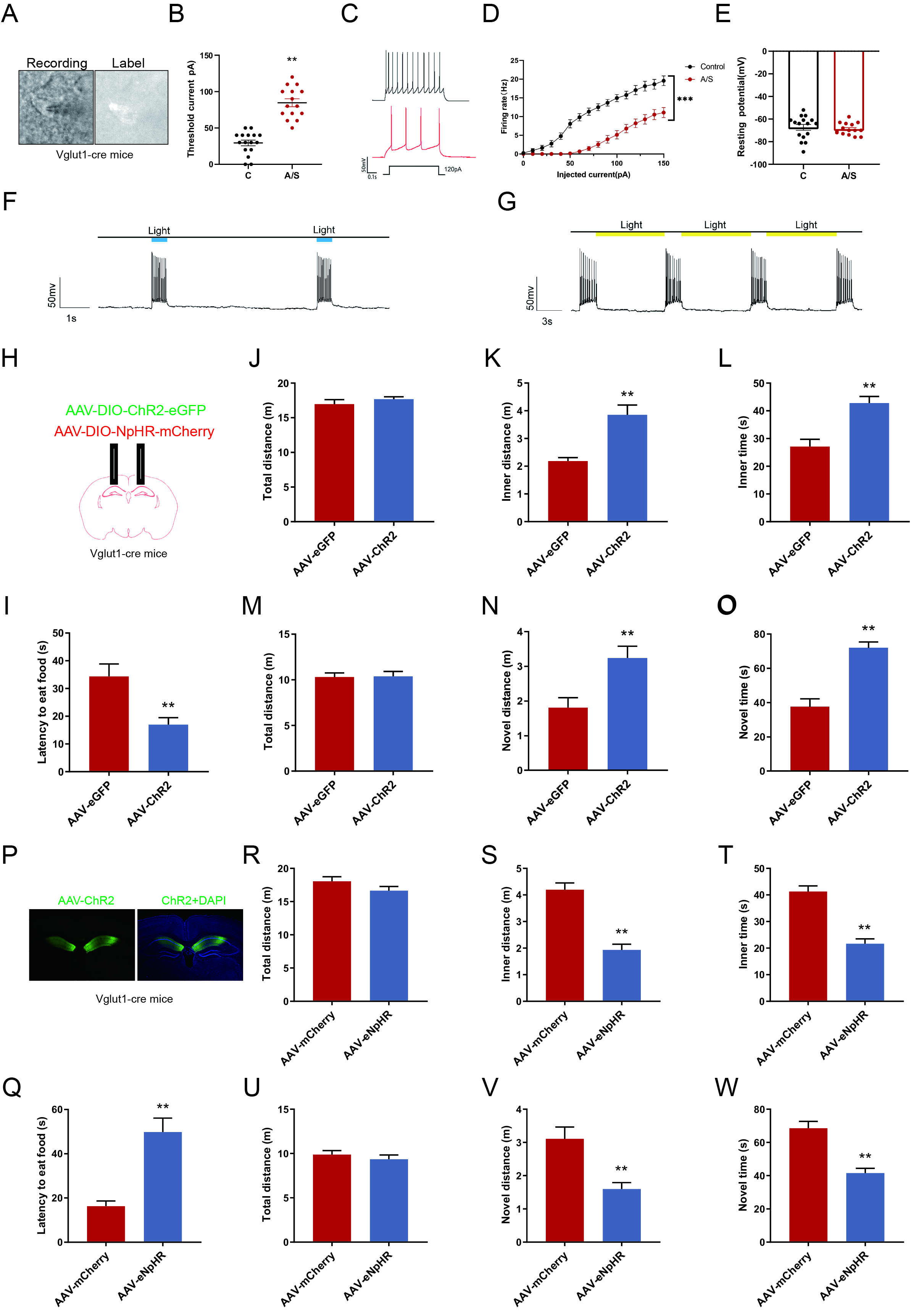

Supplement: Supplementary file 2 — Figure S1 [file CNS-30-e14902-s007.tif]

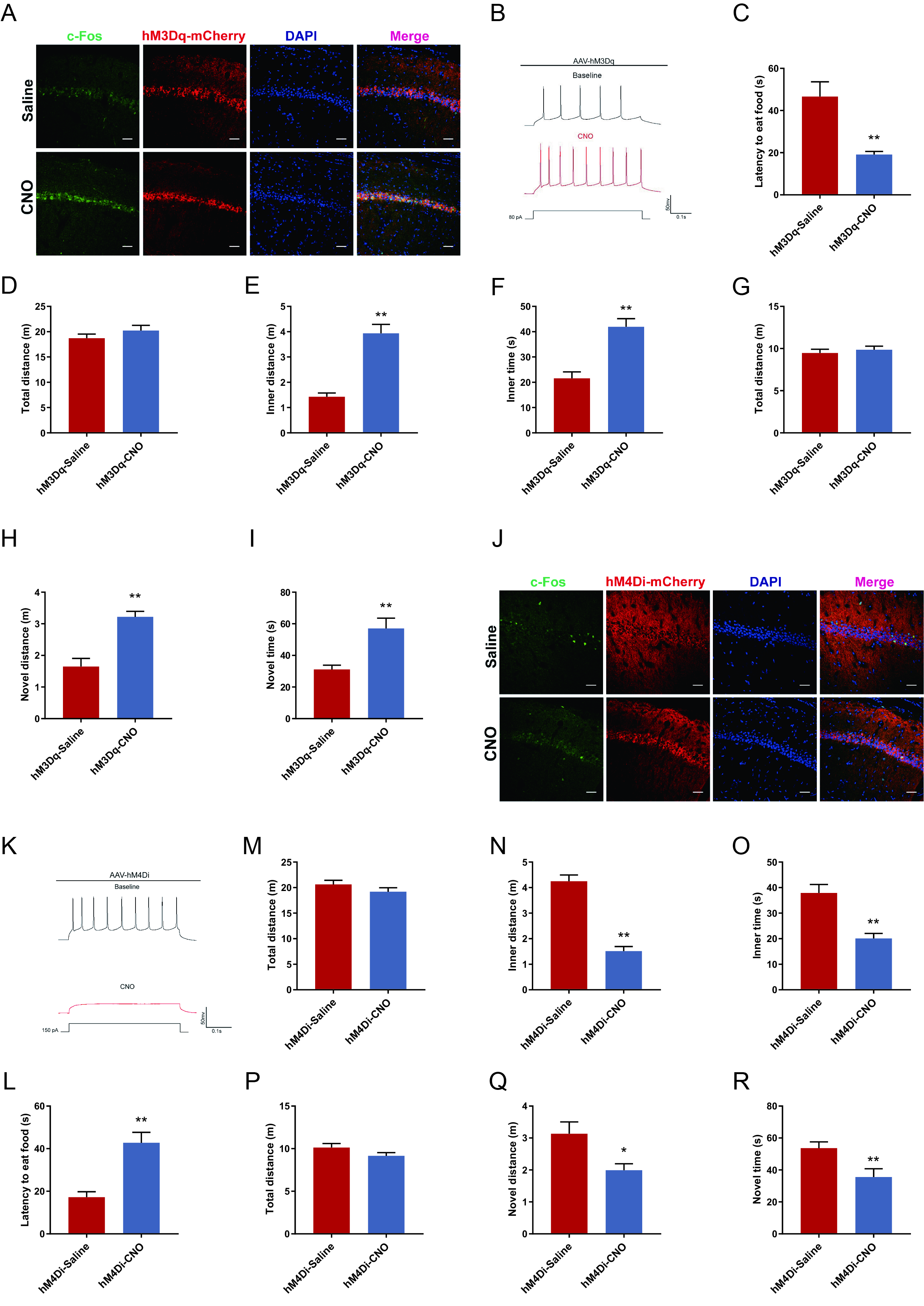

Supplement: Supplementary file 3 — Figure S2 [file CNS-30-e14902-s003.tif]

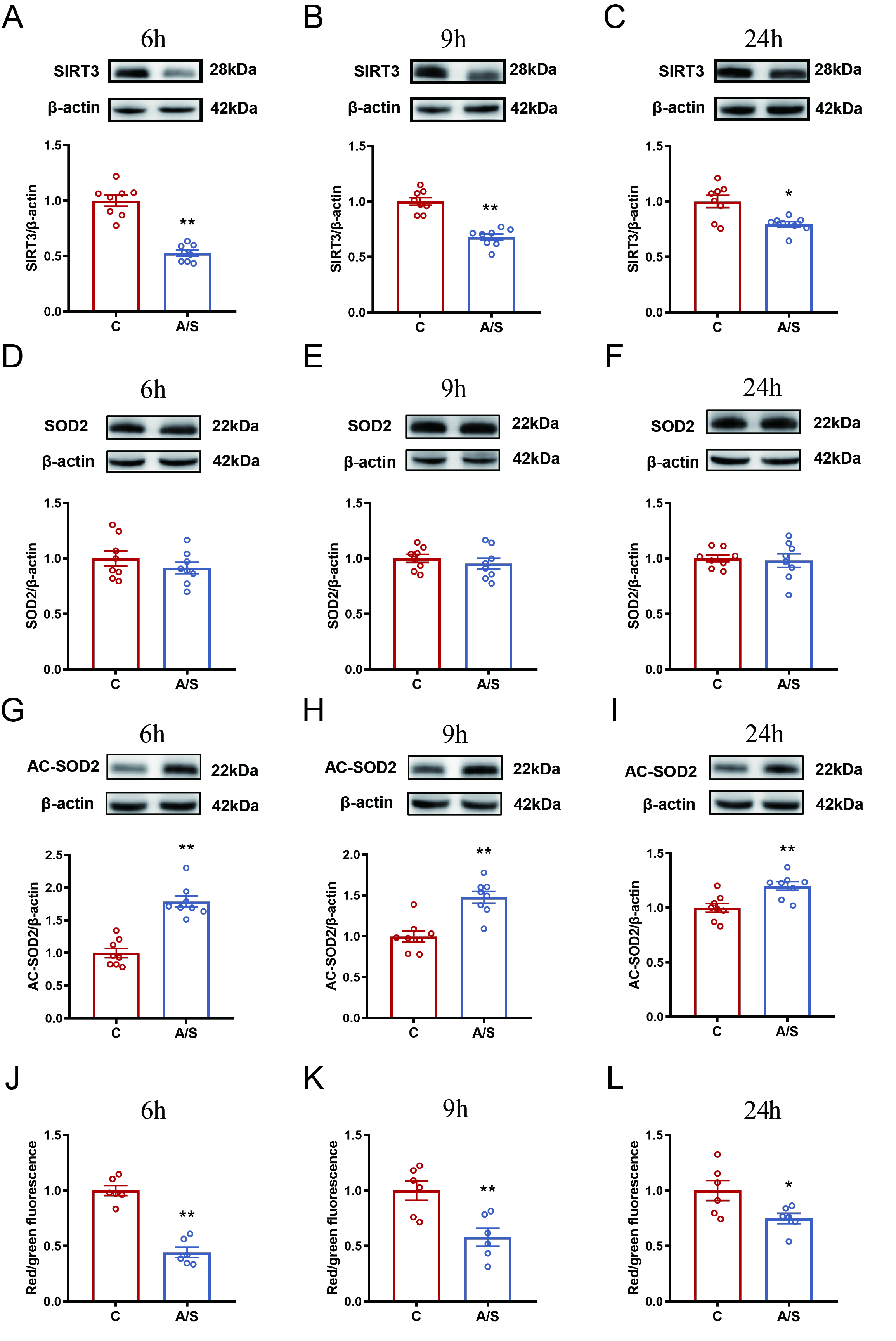

Supplement: Supplementary file 4 — Figure S3 [file CNS-30-e14902-s005.tif]

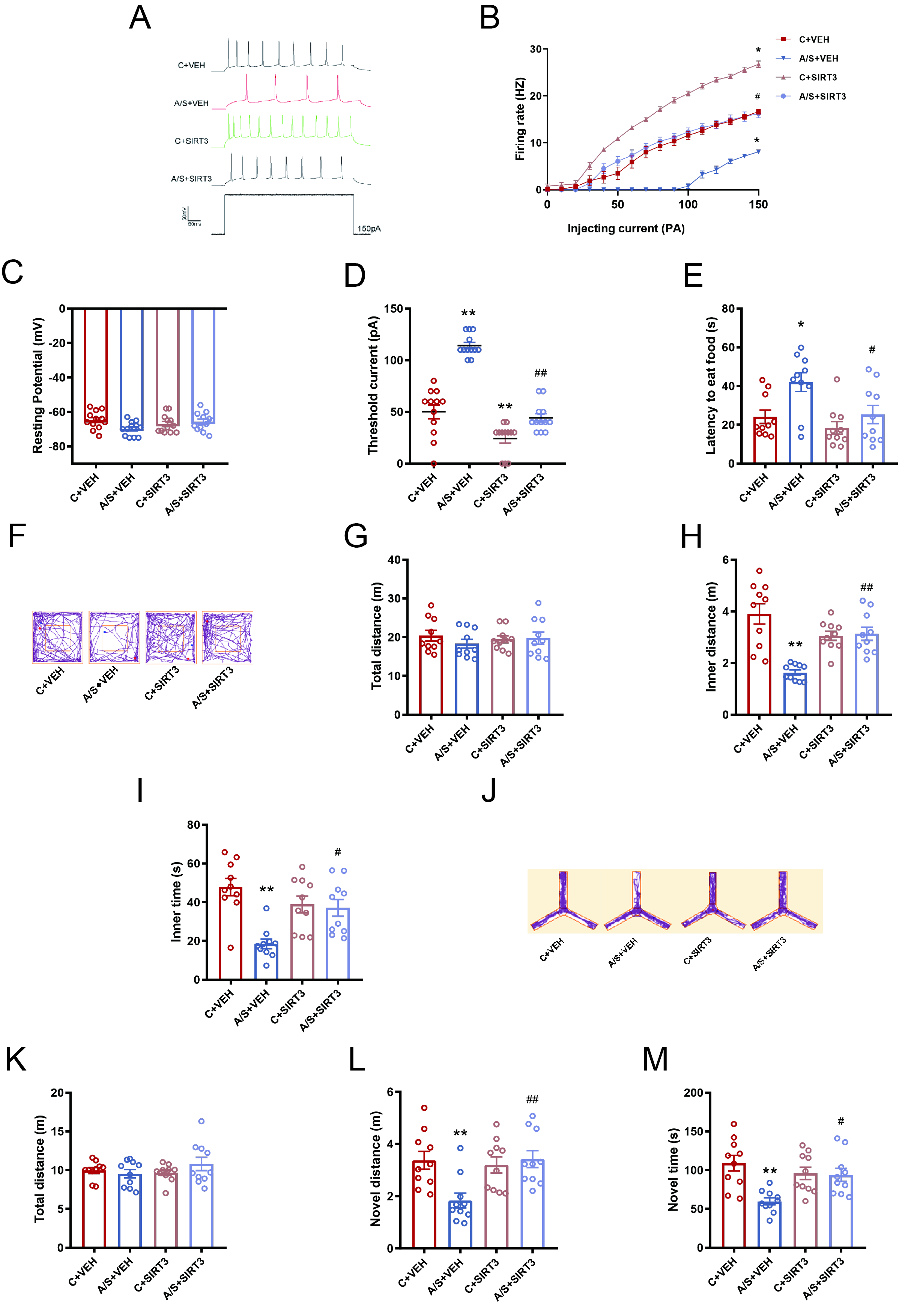

Supplement: Supplementary file 5 — Figure S4 [file CNS-30-e14902-s004.tif]

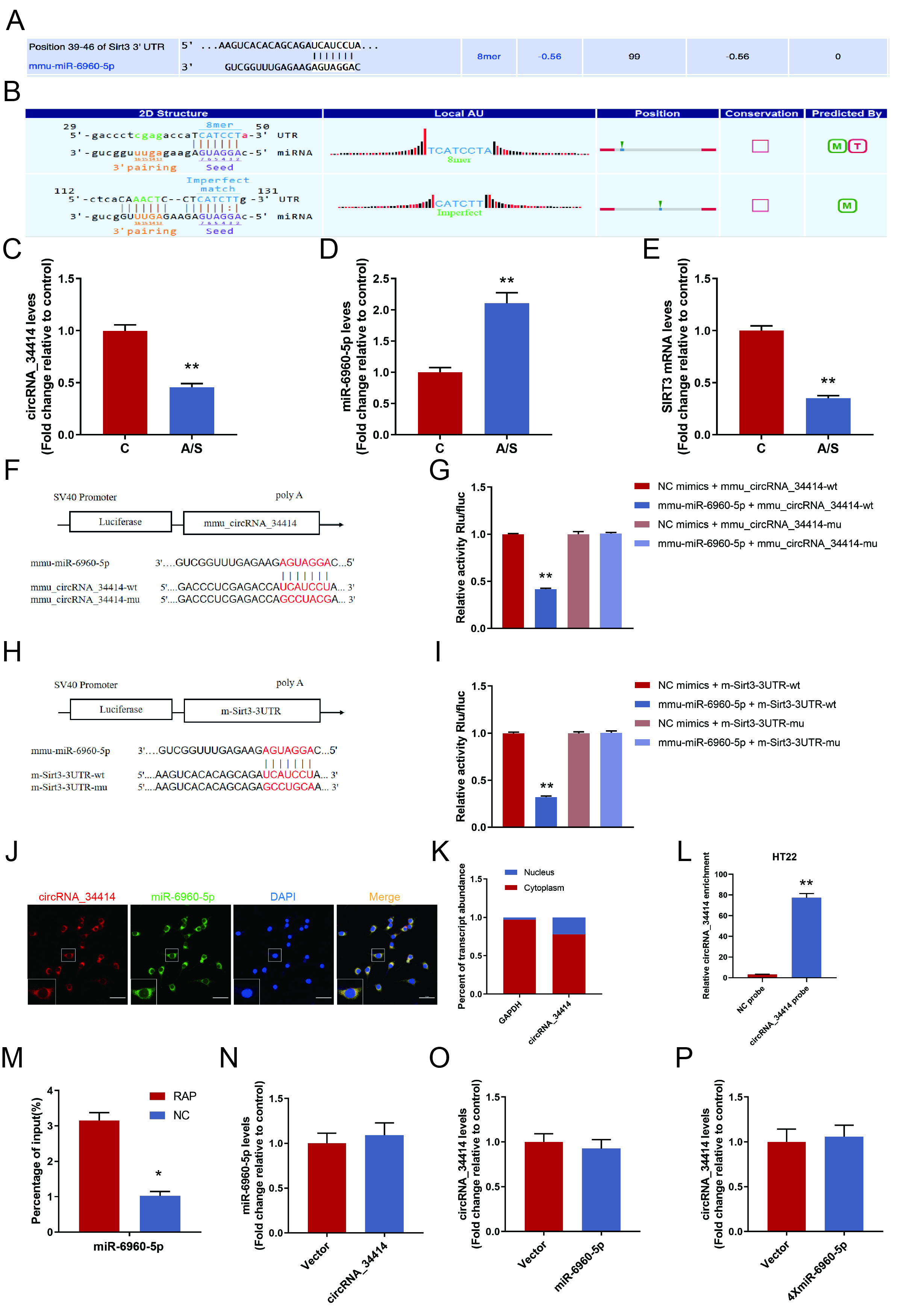

Supplement: Supplementary file 6 — Figure S5 [file CNS-30-e14902-s002.tif]
