# Supplementary material for: Role of the circRNA_34414/miR‐6960a‐5p/SIRT3 axis in postoperative delirium via CA1 Vglut1+ neurons in older mice
Source: CNS Neurosci Ther. 2024 Aug 13;30(8):e14902. doi: 10.1111/cns.14902 (PMC11322041; doi:10.1111/cns.14902)

The lanes of the unedited gel/blot that appear in the cropped image in the manuscript have been highlighted in red box.

Full unedited gel/blot for Figure 2C

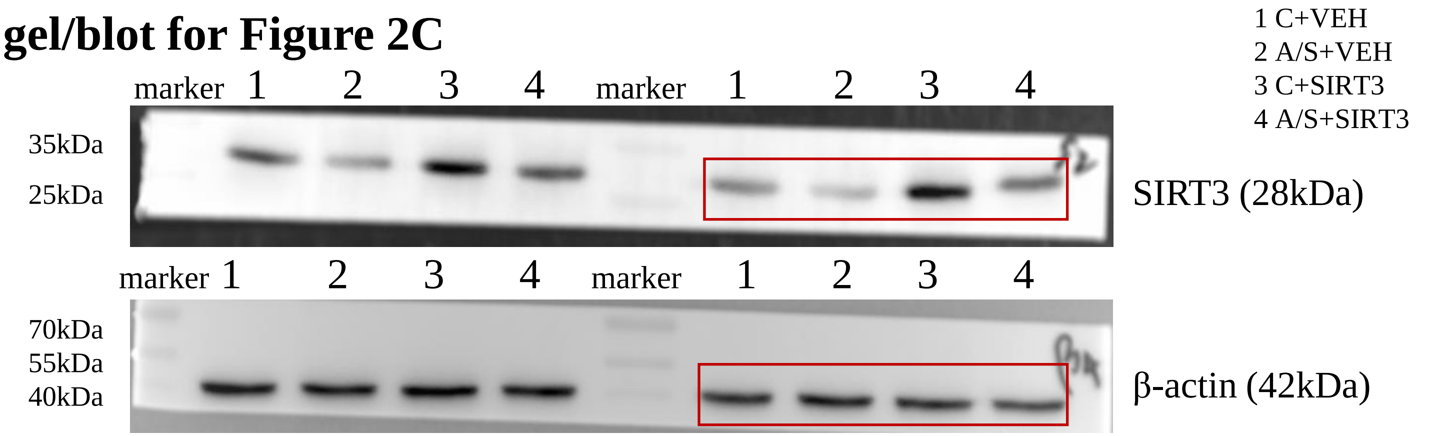

Full unedited gel/blot for Figure 2G

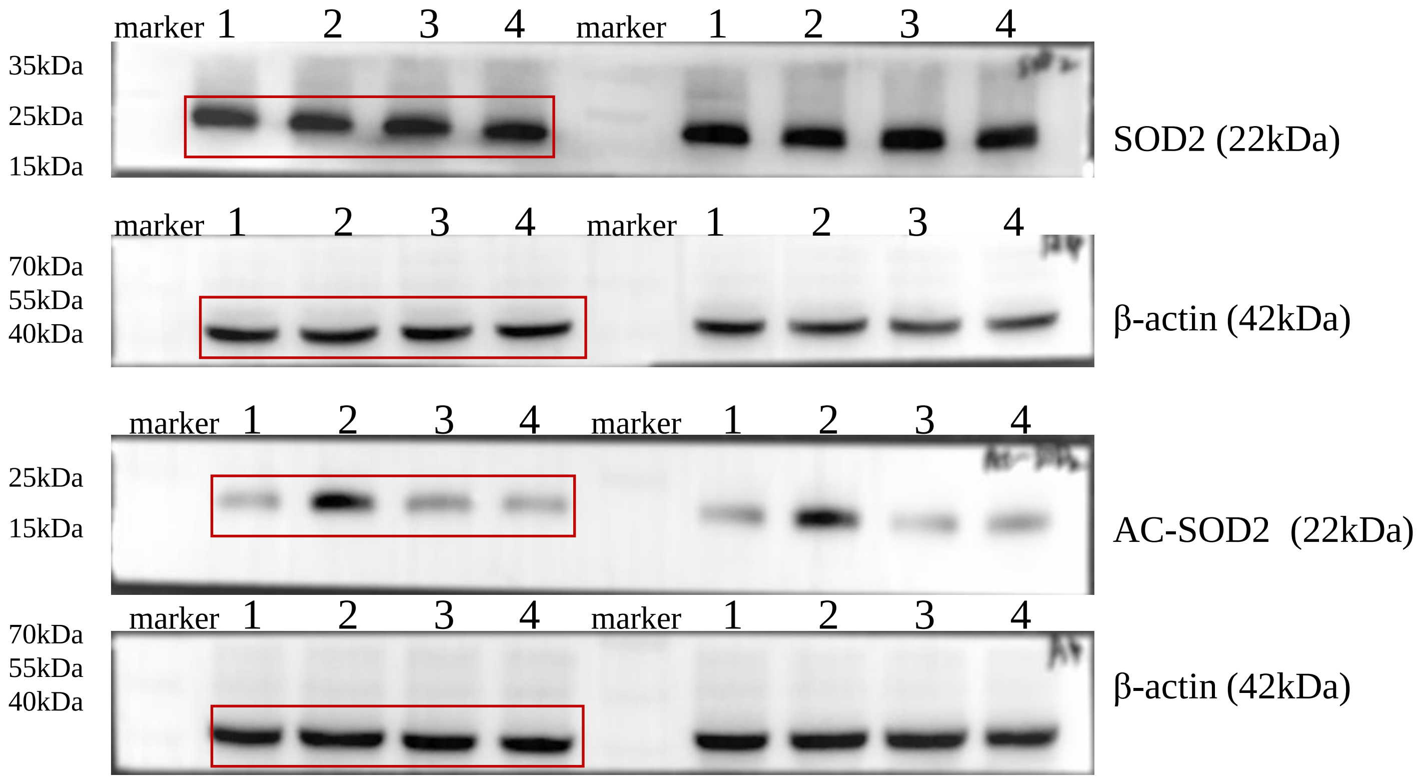

Full unedited gel/blot for Figure 3E

1 C+AAV-mCherry
2 A/S+AAV-mCherry
3 C+circRNA_34414
4 A/S+circRNA_34414

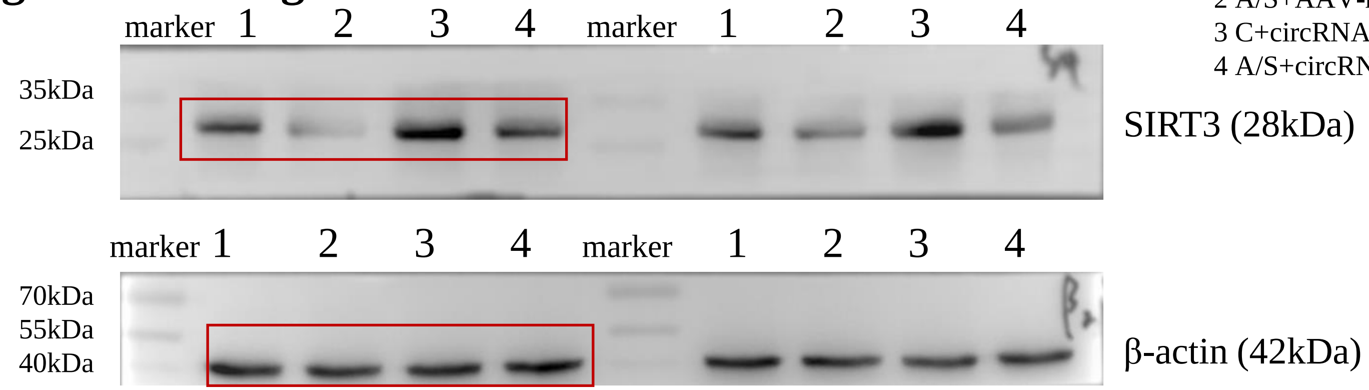

Full unedited gel/blot for Figure 3H

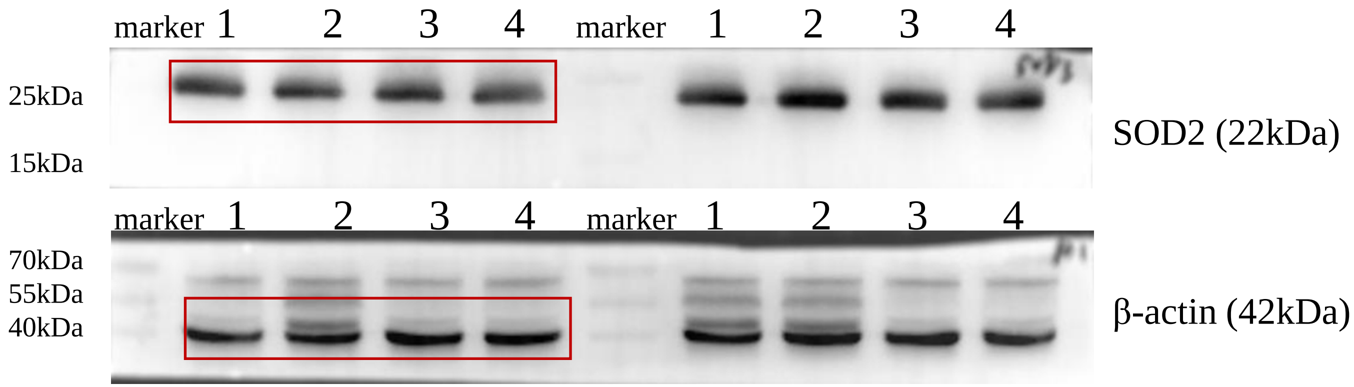

Full unedited gel/blot for Figure 3I

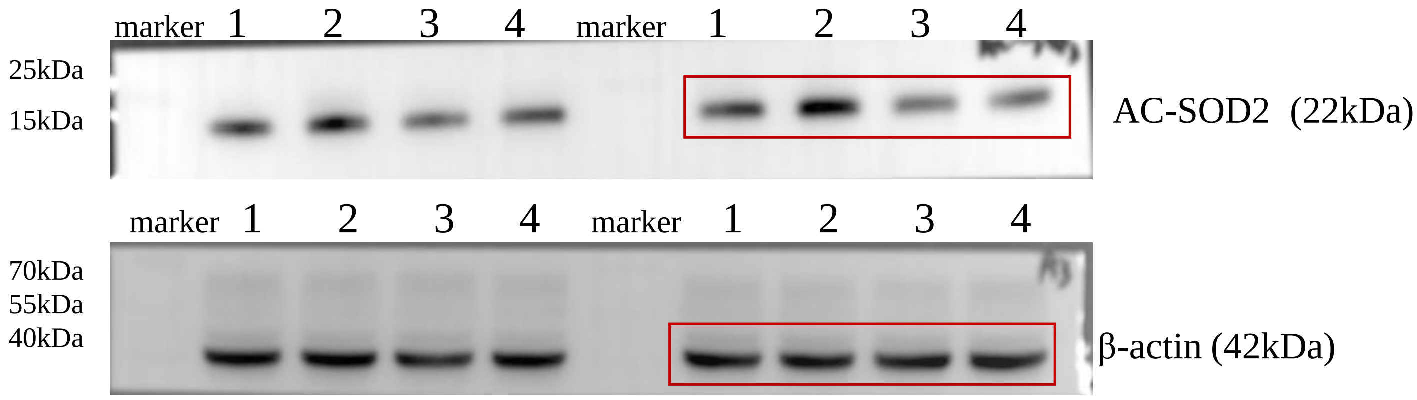

1 C+AAV-mCherry
2 A/S+AAV-mCherry
3 C+4XmiR-6960-5p
4 A/S+4XmiR-6960-5p

Full unedited gel/blot for Figure 4E

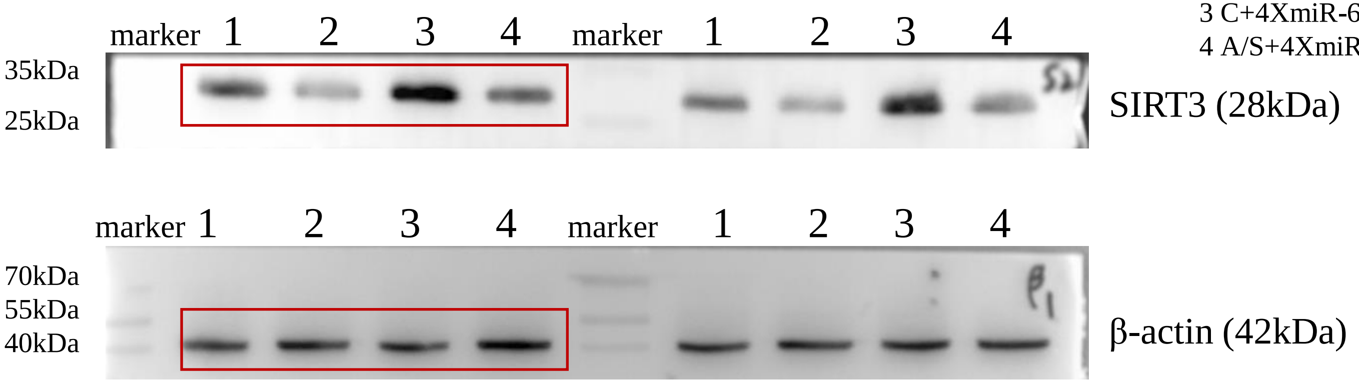

Full unedited gel/blot for Figure 4H

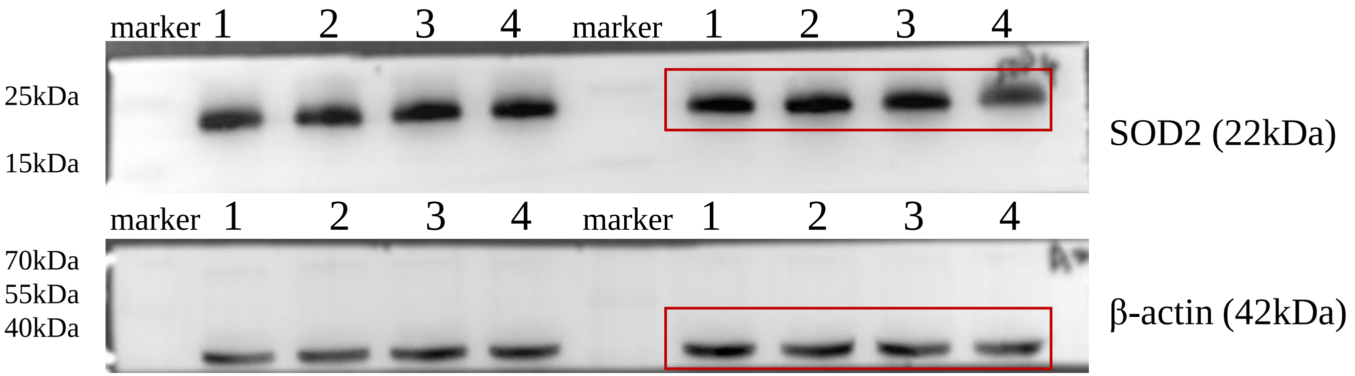

Full unedited gel/blot for Figure 4I

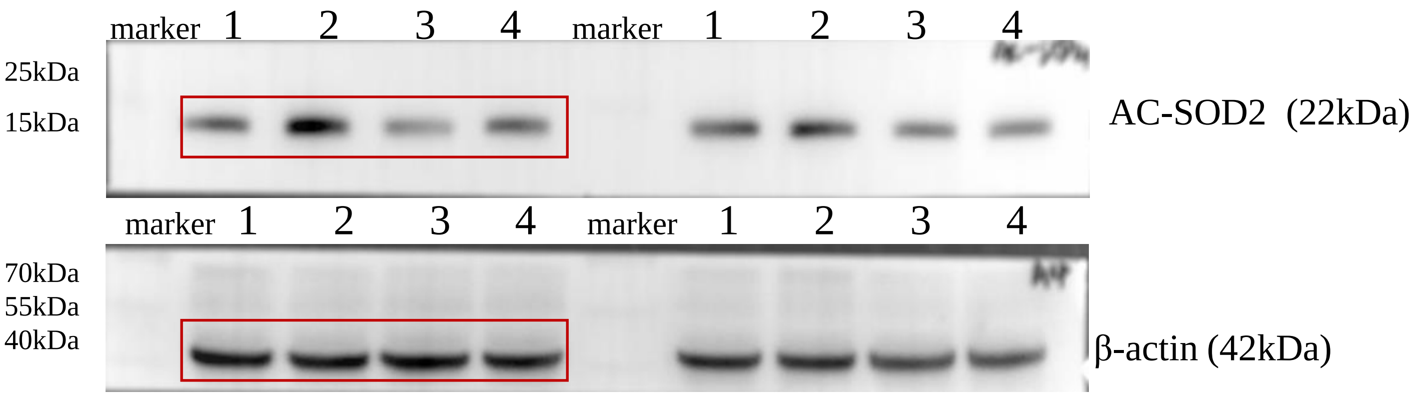

Full unedited gel/blot for Figure 5G

1 C+AAV-mCherry+AAV-BFP
2 A/S+AAV-mCherry+AAV-BFP
3 A/S+circRNA_34414+AAV-BFP
4 A/S+ circRNA_34414+miR-6960-5p

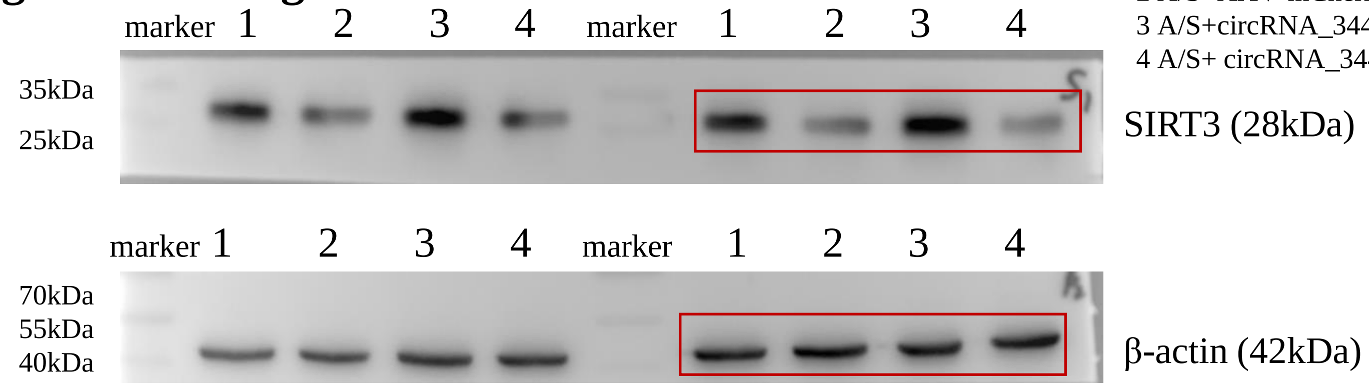

Full unedited gel/blot for Figure 5J

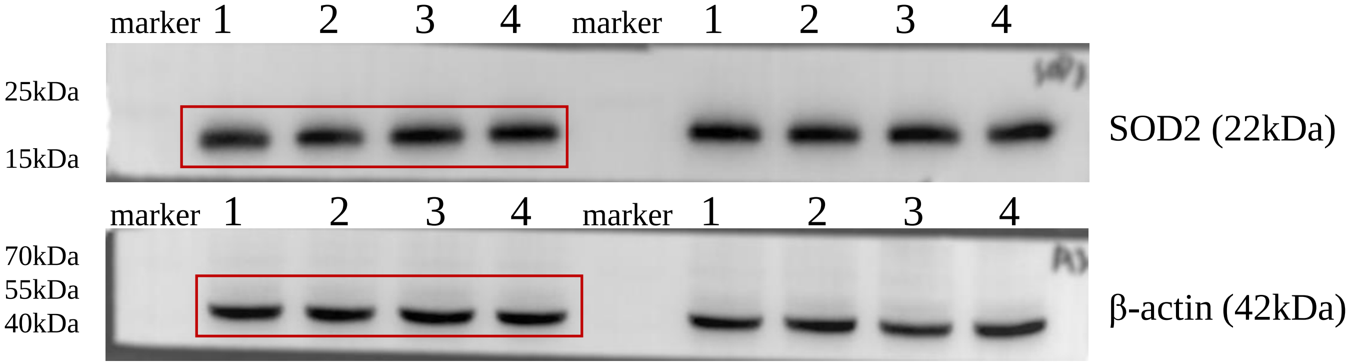

Full unedited gel/blot for Figure 5K

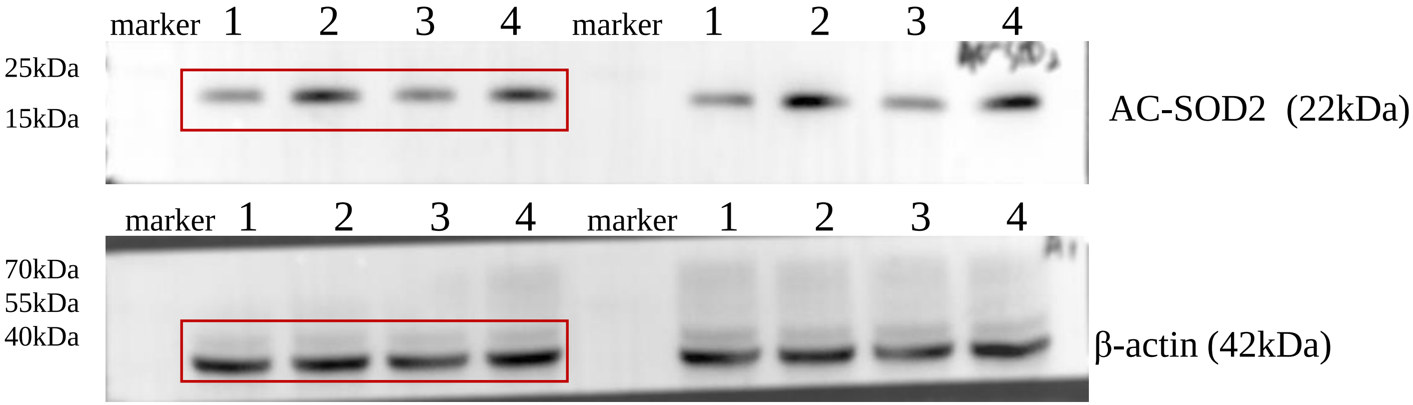

Full unedited gel/blot for Figure 6G

1 C+AAV-mCherry+AAV-BFP
2 A/S+AAV-mCherry+AAV-BFP
3 A/S+circRNA_34414+AAV-BFP
4 A/S+ circRNA_34414+SIRT3 shRNA

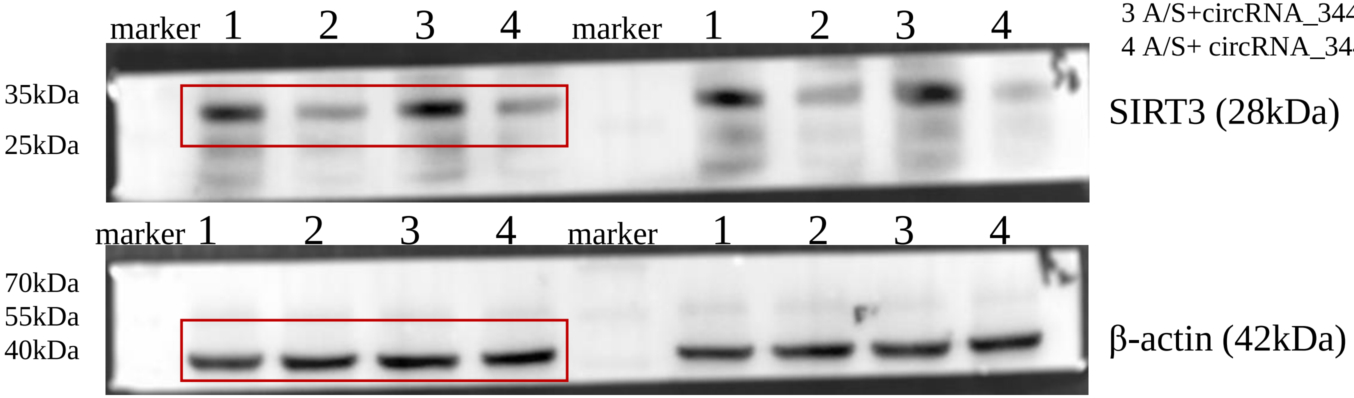

Full unedited gel/blot for Figure 6J

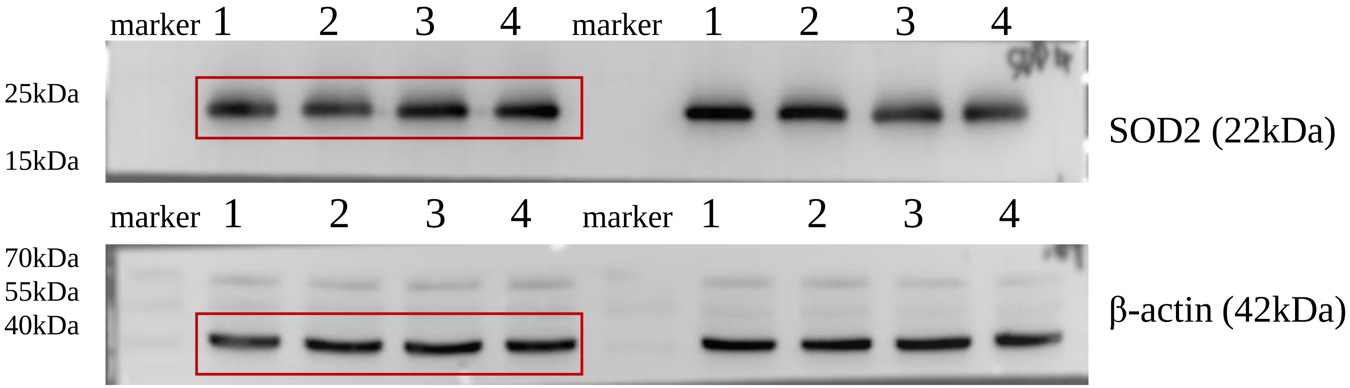

Full unedited gel/blot for Figure 6K

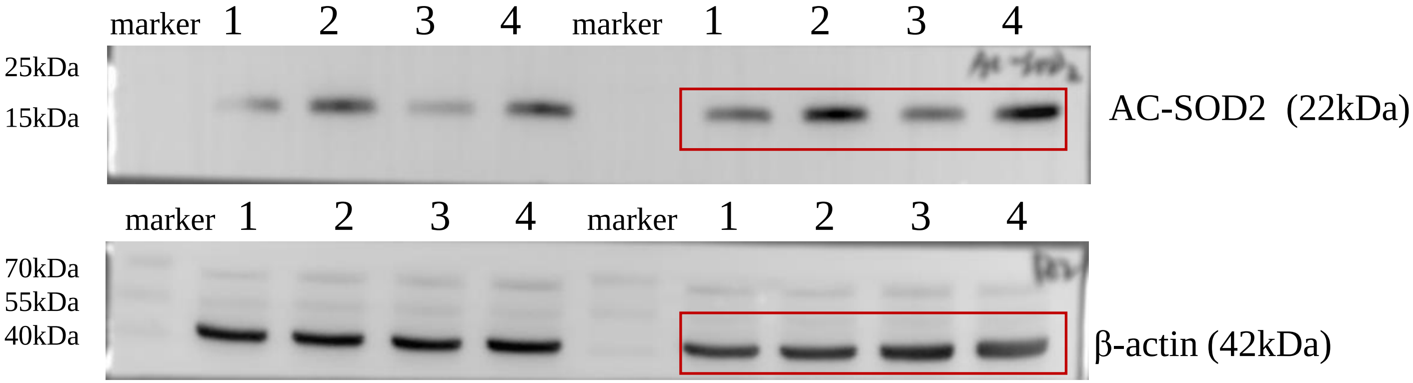

Full unedited gel/blot for Figure S3A

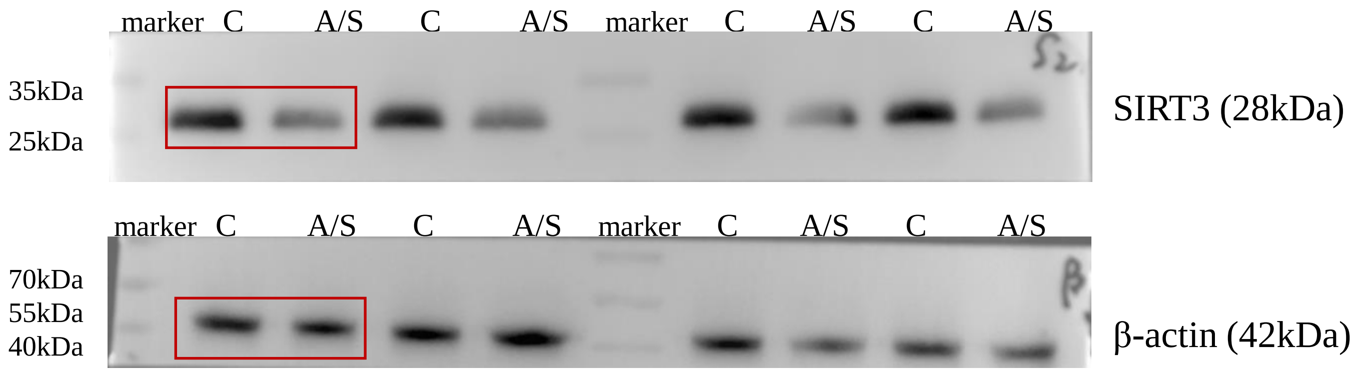

Full unedited gel/blot for Figure S3B

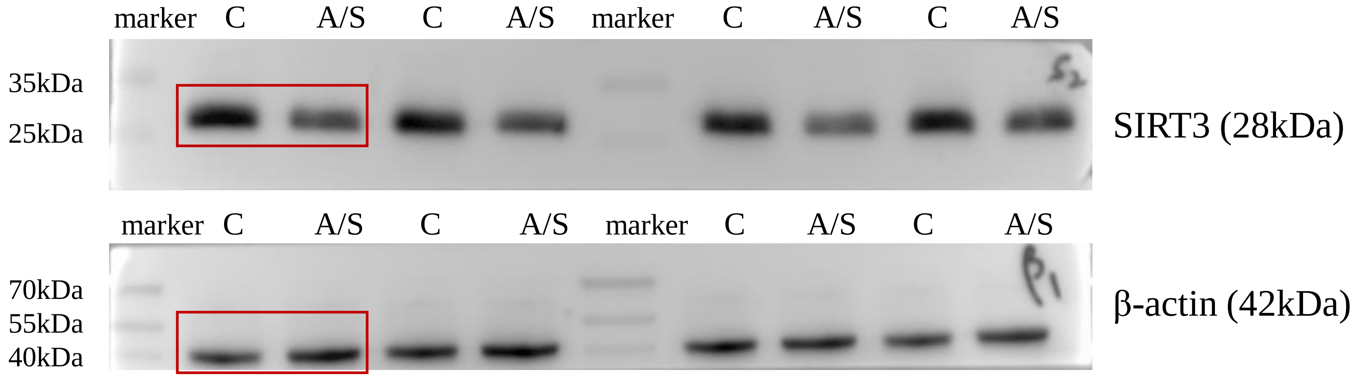

Full unedited gel/blot for Figure S3C

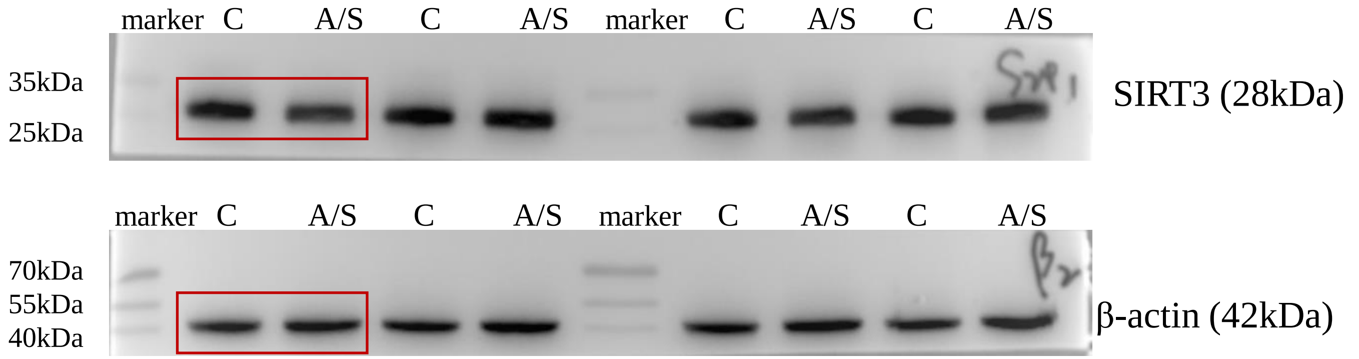

Full unedited gel/blot for Figure S3D

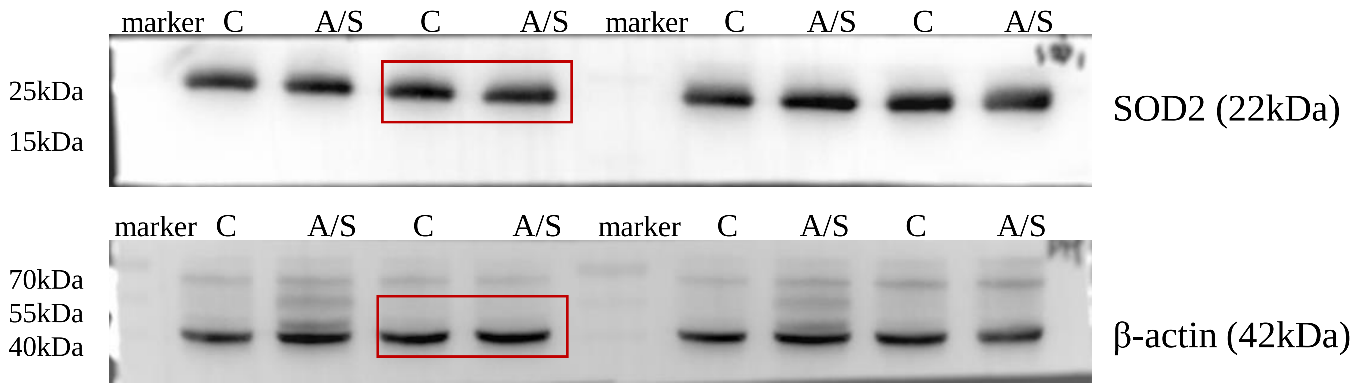

Full unedited gel/blot for Figure S3E

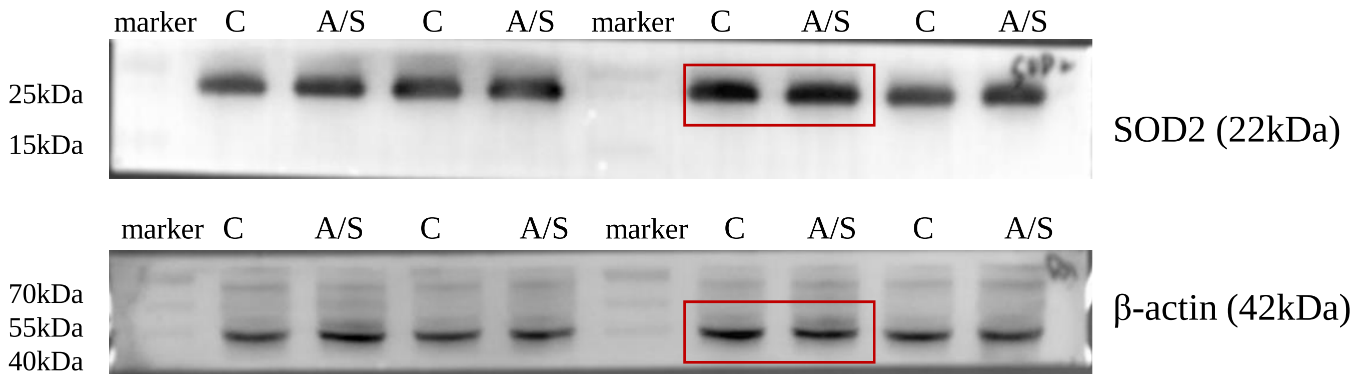

Full unedited gel/blot for Figure S3F

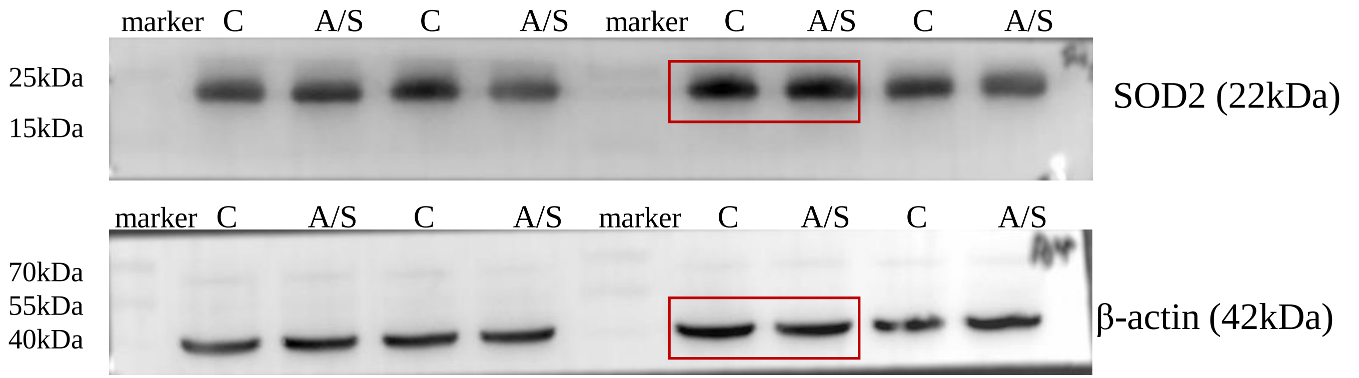

Full unedited gel/blot for Figure S3G

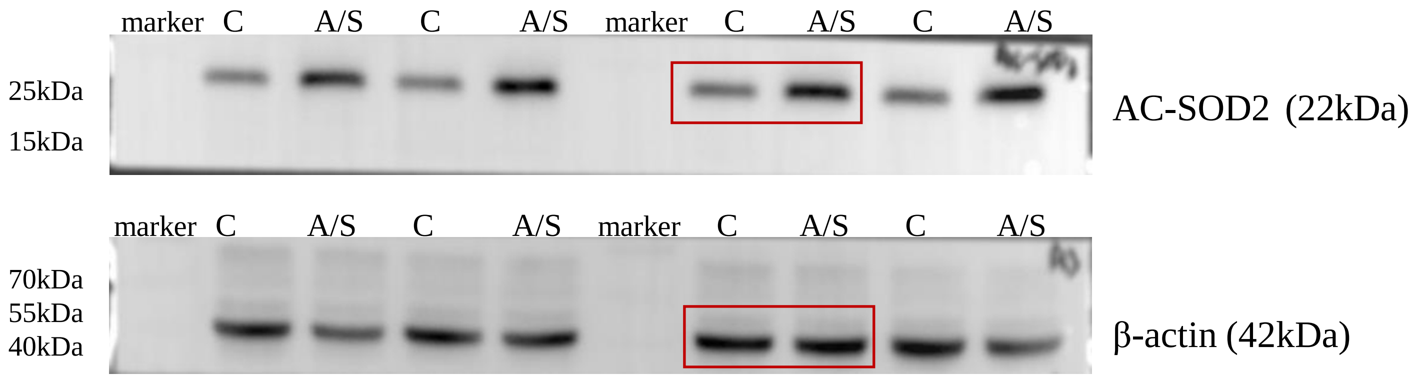

Full unedited gel/blot for Figure S3H

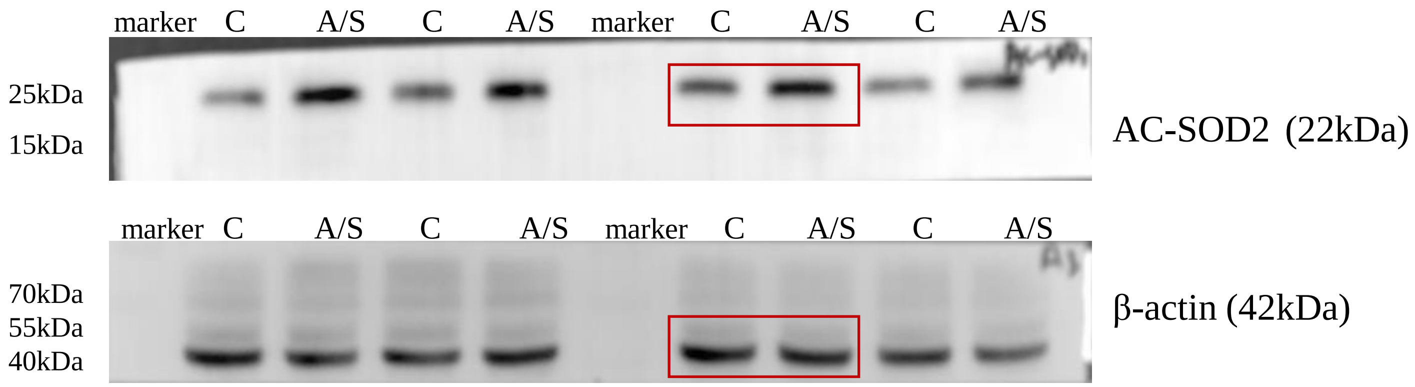

Full unedited gel/blot for Figure S3I

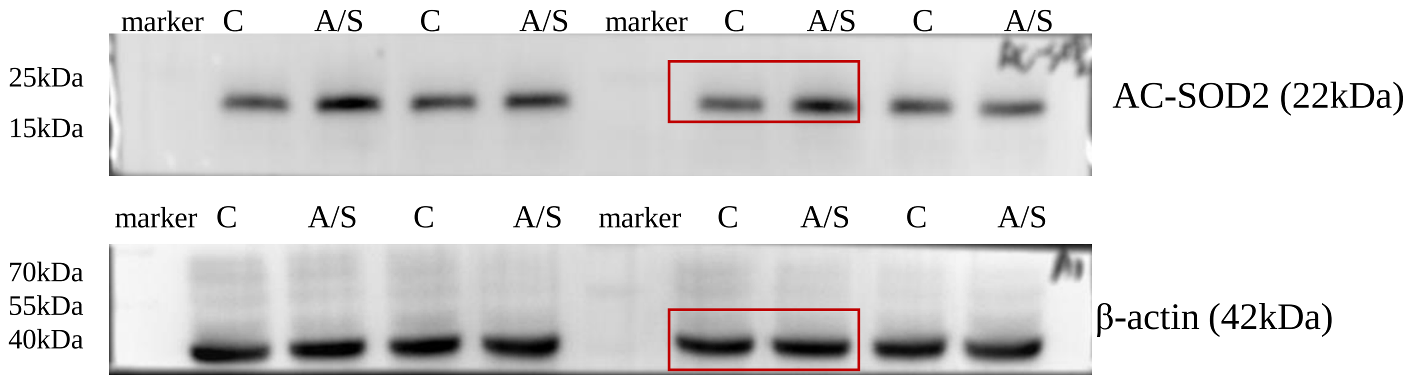

Supplement: Supplementary file 7 — Files S1 [file CNS-30-e14902-s001.pdf]
